# Female hippocampal estrogens have a significant correlation with cyclic fluctuation of hippocampal spines

**DOI:** 10.3389/fncir.2013.00149

**Published:** 2013-10-18

**Authors:** Asami Kato, Yasushi Hojo, Shimpei Higo, Yoshimasa Komatsuzaki, Gen Murakami, Hinako Yoshino, Masanao Uebayashi, Suguru Kawato

**Affiliations:** ^1^Department of Biophysics and Life Sciences, Graduate School of Arts and Sciences, The University of TokyoTokyo, Japan; ^2^Bioinformatics Project of Japan Science and Technology Agency, The University of TokyoTokyo, Japan

**Keywords:** hippocampus-synthesized steroids, estradiol, dendritic spines, estrous cycle, progesterone

## Abstract

Synaptic plasticity of the female hippocampus may cyclically fluctuate across the estrous cycle. The spine density fluctuation had been explained by fluctuation of plasma estradiol (E2) and progesterone (PROG), with the assumption that these steroids penetrate into the hippocampus. Recently, however, we demonstrated that male hippocampal levels of sex steroids are much higher than those in plasma, suggesting a weak contribution of plasma steroids to the spine density. By combination of mass-spectrometric analysis with HPLC-purification and picolinoyl-derivatization of hippocampal sex steroids, we determined the accurate concentration of E2 and PROG at four stages of plasma estrous cycle including Proestrus (Pro), Estrus (Est), Diestrus 1 (D1), and Diestrus 2 (D2). Hippocampal levels of E2 and PROG showed cyclic fluctuation with a peak at Pro for E2 and at D1 for PROG, having a positive correlation with the plasma estrous cycle. All these sex steroid levels are much higher in the hippocampus than in plasma. Even after ovariectomy a significant levels of E2 and PROG were observed in the hippocampus. The total spine density showed higher values at Pro and D1, and lower values at Est and D2, having a good correlation with the peak levels of hippocampal E2 or PROG. We also examined fluctuation of the head diameter of spines. Interestingly, mRNA expression level of steroidogenic enzymes (P450arom and 17β-HSD, etc.) and sex-steroid receptors did not significantly change across the estrous cycle. Therefore, the fluctuation of total hippocampal PROG (equal to sum of hippocampus-synthesized PROG and plasma PROG) may be originated from the contribution of cyclic change in plasma PROG, which can induce the fluctuation of total hippocampal E2, since steroid conversion activity of hippocampus might be nearly the same across the estrus cycle.

## Introduction

Sex steroids play an indispensable role in the regulation of the neural properties in the brain (Hajszan et al., 2007; McCarthy, 2008). For example, 17β-estradiol (E2), converted from testosterone (T), induces sexual dimorphism in the preoptic area and the ventromedial nucleus of the hypothalamus (Gorski et al., 1978; Davis et al., 1996). The hippocampus is a target for neuromodulatory actions of sex hormones.

*In vivo* supplementation with estrogen enhances the hippocampus-related learning scores of ovariectomized (OVX) female rats (Luine et al., 2003; Sandstrom and Williams, 2004; Sinopoli et al., 2006) or monkeys (Rapp et al., 2003), and rescues the density of dendritic spines in CA1 region of the hippocampus in OVX female rodents or primates (Gould et al., 1990; Woolley and McEwen, 1993; HaoJ et al., 2003; Tang et al., 2004; MacLusky et al., 2005). *In vitro* E2 supplementation to male hippocampal slices increases the density of CA1 spines and enhances the long-term depression (LTD) of adult rats (Mukai et al., 2007).

In female rats, the circulating female sex steroid levels are largely altered depending on the estrus cycle composed of 4 stages, including Proestrus (Pro), Estrus (Est), Diestrus 1 (D1), and Diestrus 2 (D2) (the stage duration is 1 day each) (Watanabe et al., 1990). Plasma E2 level shows a peak at Pro, is reduced at Est and D1, restored to some extent at D2, and increased at Pro. After the ovulation, plasma progesterone (PROG) level shows a peak at D1.

Many studies report the estrous cycle-dependent changes in the female hippocampal synaptic plasticity, including the density of spines (Woolley and McEwen, 1992; Bi et al., 2001; Markham et al., 2005), the magnitude of long-term potentiation (LTP) (Good et al., 1999; Warren et al., 1995) and the performance of hippocampus-dependent tasks such as spatial learning (Warren and Juraska, 1997) and object recognition test (Walf et al., 2006).

These female hippocampal changes are explained as the consequence of the changes in the level of circulating E2 or PROG which is assumed to penetrate into the hippocampus. Recent studies, however, show that adult male rat hippocampal neurons synthesize estrogen and androgen whose levels are higher than those in plasma (Kawato et al., 2002; Hojo et al., 2004, 2008; Okamoto et al., 2012). *In vivo* inhibition of E2 synthesis by letrozole injection suppressed LTP-induction in female hippocampus (Vierk et al., 2012). Therefore, the female hippocampal levels of E2 and PROG in each stage of cycle should be determined to answer several questions; for example, (1) are sex steroid levels in the hippocampus much higher than those in plasma? or (2) are hippocampal sex steroid levels cyclically fluctuating ?

Here, we investigate whether the levels of sex steroids, steroidogenic enzymes and sex steroid receptors change across the estrous cycle. We determine the accurate concentration of sex steroids in the hippocampus by using liquid chromatography-tandem mass spectrometry (LC-MS/MS).

## Materials and methods

### Animals

Wistar rats (10 weeks old) were purchased from Saitama Experimental Animals Supply (Japan). The estrous cycle of female rats was monitored with morning vaginal smears. Only those rats showing three consecutive 4-day cycles of Pro, Est, D1, and D2 were used at the age of 12 weeks old. OVX and sham operations were performed 2-weeks before (at 10 weeks old) the experiments. For estradiol replacement, OVX rats received subcutaneous injection of E2 (40 μg/kg body weight) dissolved in sesame oil, 5 h before hippocampal E2 measurements. Male rats were also used at the age of 12 weeks old. All animals were maintained under a 12 h light/12 h dark exposure and free access to food and water. The experimental procedure of this research was approved by the Committee for Animal Research of the University of Tokyo.

### Chemicals

E2, T, estrone (E1), androstenedione (ADione), and progesterone (PROG) were purchased from Sigma (USA). Picolinic acid was from Tokyo Chemical Industry (Japan) and [1,2,3,4-^13^C_4_]E2 and [1,2,3,4-^13^C_4_]E1 were from Hayashi Pure Chemical (Japan). T-d_3_, ADione-d_7_, PROG-d_4_ were from CDN Isotope Inc. (Canada). [^3^H] labeled steroids ([2,4,6,7-^3^H]-E2, [1,2,6,7-^3^H]-T, [2,4,6,7-^3^H]-E1, [1,2,6,7-^3^H]-PROG, and [1,2,6,7-^3^H]-ADione) were purchased from Perkin Elmer (USA).

### Mass-spectrometric assay of steroids

Detailed procedures are described elsewhere (Hojo et al., 2009).

#### Step (1) purification of steroids from hippocampi with normal phase HPLC

A rat was deeply anesthetized and decapitated at 10–10:30 a.m., since at this time window E2 surge at Pro occurs in plasma (Gorski et al., 1975). The whole hippocampi was removed and homogenized. To calculate the recovery of steroids, radioactive steroids (20,000 cpm) were added as internal standards to hippocampal homogenate. To extract steroid metabolites, ethyl acetate/hexane (3:2 vol/vol) was applied to the homogenates which were then mixed. The mixture was centrifuged at 2500 × g and the organic layer was collected. After evaporation, the extracts were dissolved in 1 ml of 40% methanol/H_2_O and applied to a C_18_ Amprep solid phase column (Amersham Biosciences, USA) to remove contaminating fats. The extracts were dried, dissolved in an elution solvent of HPLC. The steroid metabolites were separated into PROG, E1, E2, ADione, and T using a normal phase HPLC system (Jasco, Japan) with an elution solvent of hexane: isopropylalcohol: acetic acid = 98:2:1. A silica gel column (0.46 × 15 cm, Cosmosil 5SL, Nacalai Tesque, Japan) was used.

By monitoring ^3^H-steroids, the recoveries of PROG, E1, E2, ADione, and T and were 44 ± 3, 43 ± 2, 46 ± 4, 47 ± 4, and 49 ± 4%, respectively, after extraction, C_18_ column treatment and normal phase HPLC separation. Purification of steroids from the blood plasma was also performed. Plasma was prepared by centrifugation at 1900 × g, 4°C for 15 min of trunk blood collected from the same rats used for the measurements of hippocampal steroids. Extraction of sex steroids was performed by centrifugation at 2500 × g of the mixture of plasma with 100% ether, and the organic layer was collected and evaporated. As internal standards, 100 pg of isotope labeled steroids (PROG-d_4_, ^13^C_4_-E1, ^13^C_4_-E2, ADione-d_7_, and T-d_3_) were added to steroid extracts.

#### Step (2) derivatization of HPLC-purified steroids before application to LC (reverse phase)-MS/MS

Preparation and purifiation of E2-pentafluorobenzyl (PFBz)-17-picolinoyl-ester, T-17-picolinoyl-ester, E1-picolinoyl-ester were performed with slight modification of previous methods (Hojo et al., 2009). For preparation of E2-PFBz-picolinoyl, evaporated E2 extracts from the hippocampus or evaporated total steroid extracts from plasma, were reacted with 150 μL of reaction reagent (2.5% pentafluorobenzyl bromide/acetonitrile: 0.8% KOH/ethanol = 2:1) at 53°C for 1 h. After evaporation, the products were reacted with 50 μl of picolinoic acid suspension (4% picolinoic acid, 4% of 4-dimethylaminopyridine, 2% 2-methyl-6-nitrobenzoic anhydride in tetrahydrofuran anhydrous) (i.e., 80 mg of picolinoic acid, 80 mg of 4-dimethylaminopyridine, 40 mg of 2-methyl-6-nitrobenzoic acid in 2 mL of tetrahydrofuran) and 20 μL of triethylamine, for 0.5 h at room temperature. The reaction products dissolved in 1% acetic acid were purified using a Bond Elute C_18_ column (Varian, USA). The dried sample was dissolved in elution solvent of LC. For preparation of E1-3-picolinoyl-ester and T-17-picolinoyl-ester, evaporated steroid extracts from the hippocampus or plasma were reacted with 50 μl of picolinoic acid suspension (4% picolinoic acid, 4% of 4-dimethylaminopyridine, 2% 2-methyl-6-nitrobenzoic anhydride in tetrahydrofuran anhydrous) and 20 μL of triethylamine, for 0.5 h at room temperature. The reaction products were purified with the C_18_ column by using 80% acetonitrile. The purified E1 or T-derivative was dissolved in elution solvent of LC. For PROG and ADione, derivatization was not performed.

#### Step (3) determination of the concentration for PROG, E1, E2, ADione, and T using LC-MS/MS

For determination of the concentration of all the steroids, the LC-MS/MS system, which consists of a Shimadzu HPLC system and an API-5000 triple stage quadrupole mass spectrometer (Applied Biosystems, USA) were employed. LC chromatographic separation was performed on a Cadenza CD-C_18_ column (3 × 150 mm, 3 μm, Imtakt Japan). MS analysis was operated with electro spray ionization (ESI) in the positive-ion mode. The isotope-labeled steroid derivatives were used for calibration of retention time by monitoring the m/z transition, from 318 to 100 for PROG-d_3_, from 380 to 161 for E1-^13^C_4_-picolinoyl, from 562 to 343 for E2-^13^C_4_-PFBz-picolinoyl, from 294 to 113 for ADione-d_7_, and from 397 to 256 for T-d_3_-picolinoyl-ester, respectively. Isotope-labeled steroid derivatives were used for internal standards in order to measure recovery of steroids as well as to calibrate the retention time. By monitoring isotope steroids, the recoveries of PROG, E1, E2, ADione, and T were determined as 72 ± 4, 75 ± 5, 86 ± 2, 73 ± 4, and 74 ± 4%, respectively, after derivatization, purification and MS/MS detection.

In the multiple reaction monitoring mode, the instrument monitored the m/z transition, from 315 to 97 for PROG, from 376 to 157 for E1-picolinoyl, from 558 to 339 for E2-PFBz-picolinoyl, from 287 to 109 for ADione, and from 394 to 253 for T-picolinoyl, respectively, Figure S1; Table S1). Here, m and z represent the mass and charge of a steroid derivative, respectively.

The limits of quantification for steroids were measured with blank samples, prepared alongside hippocampal samples through the whole extraction, fractionation, and purification procedures. The limits of quantification for PROG, E1, E2, ADione, and T were 2, 1, 0.3, 2, and 1 pg per 0.1 g of hippocampal tissue or 1 mL of plasma, respectively (Table S1).

From the calibration curve using standard steroids dissolved in blank samples, the linearity was observed between 2 and 1250 pg for PROG and ADione, between 1 and 1000 pg for E1, between 0.1 and 1000 pg for E2, and between 0.5 and 1000 pg for T, respectively (Figure S2).

### RT-PCR and southern blotting

The detailed procedures of mRNA analyses are described in elsewhere (Kawato et al., 2002; Hojo et al., 2004; Kimoto et al., 2010). Total RNAs were isolated from adult rat tissues such as hippocampus, ovary, and testis, using a SV total RNA Isolation System (Promega, USA). The purified RNAs were treated with RNase-free DNase to eliminate the possibility of genomic DNA contamination, and quantified on the basis of the absorbance at 260/280 nm. The purified RNAs (100 ng) were reverse-transcribed to obtain cDNAs, using a M-MLV Reverse Transcriptase (Promega, USA). PCR was performed by using these cDNAs. The oligonucleotides for PCR amplification were designed as illustrated in Table S2. The PCR protocols comprised application of a 30 s denaturation period at 95°C, a 20 s annealing period at individual temperature for each enzyme, and a 30 s extension at 72°C, for individual number of cycles for each enzyme (Table S2). For semi-quantitative analysis, the RT-PCR products were separated on 2% agarose gels, stained with ethidium bromide, and analyzed with a fluorescence gel scanner (Atto, Japan) and Image J software. In all the cases we first plotted amplification curves in order to choose the linear phase of PCR cycles (Figure S3). The semi-quantitative comparison between different estrus cycle stages can be performed after normalization by glyceraldehyde-3-phosphate dehydrogenase (GAPDH, a house keeping gene) as internal standard. The optimal cycle number of GAPDH mRNA was determined as 17 from the amplification curve. The comparison of relative abundance between different estrous stages or between female and male was performed by using the normalized expression. Note that GAPDH expression was not changed across the estrus cycle. As positive control, we used the reference organ (ovary or testis). To further confirm the expression, Southern hybridization was performed. The amplified RT-PCR products were directly cloned into TA-cloning vector (Promega, USA), and sequenced. The resulting sequence was identical to the reported cDNA sequences of these enzymes. These cloned products were used as the template of DNA probes for Southern hybridization. After transfer of the RT-PCR products from agarose gels to nylon membrane (Hybond N+, Amersham, USA), Southern hybridization was performed with Fluorescein-labeled cDNA probes for these enzymes and GAPDH using a Gene Image Random Prime Labeling Module (GE healthcare, USA). The Southern hybridization signals were then measured using a LAS-3000 Image analyzer (Fuji film, Japan).

### Imaging and analysis of dendritic spine density and morphology

#### Slice preparation (perfusion-fixed slices)

Hippocampal slices were prepared from a 12 week-old female or male rat deeply anesthetized and perfused transcardially with PBS (0.1 M phosphate buffer and 0.14 M NaCl, pH 7.3), followed by fixative solution of 3.5% paraformaldehyde. Decapitation was performed at 10:00–10:30 a.m., since we identified estrous stage at this time window. Immediately after decapitation, the brain was removed from the skull and post-fixed with fixative solution. Hippocampal slices, 400 μm thick, were prepared with a vibratome (Dosaka, Japan).

#### Slice preparation (acute slices)

Twelve weeks male rats were deeply anesthetized. Without paraformaldehyde fixation procedures, decapitation was performed. Immediately after decapitation, the brain was removed from the skull and placed in ice-cold oxygenated (95% O_2_, 5% CO_2_) artificial cerebrospinal fluid (ACSF) containing (in mM): 124 NaCl, 5 KCl, 1.25 NaH_2_PO_4_, 2 MgSO_4_, 2 CaCl_2_, 22 NaHCO_3_, and 10 D-glucose (all from Wako); pH was set at 7.4. Hippocampal slices, 400 μm thick, were prepared with a vibratome (Dosaka, Japan). These slices were “fresh” slices without ACSF incubation. Slices were then incubated in oxygenated ACSF for 2 h (slice recovery processes) in order to obtain widely used “acute slices.” These “acute” slices were then incubated at room temperature with E2 or PROG. Then, slices were prefixed with 4% paraformaldehyde at 4°C for 4 h.

#### Current injection of neurons by Lucifer Yellow

Neurons within slices were visualized by an injection of Lucifer Yellow under a Nikon E600FN microscope (Japan) equipped with a C2400–79H infrared camera (Hamamatsu Photonics, Japan) and with a 40 × water immersion lens (Nikon). A glass electrode was filled with 5% Lucifer Yellow, which was then injected for 15 min using Axopatch 200B (Axon Instruments, USA). Approximately five neurons within a 100–200 μm depth from the surface of a slice were injected (Duan et al., 2002).

#### Confocal laser microscopy and morphological analysis

Spine imaging and analysis with confocal microscopy were performed as described previously (Komatsuzaki et al., 2005; Tsurugizawa et al., 2005; Mukai et al., 2006, 2011). We analyzed the spines of CA1 pyramidal neurons, along apical dendrites in stratum radiatum of the dorsal hippocampus. The branch order is secondary branch with 100–250 μm distance from the soma (Murakami et al., 2006; Mukai et al., 2007; Komatsuzaki et al., 2012). CA2 and CA3 were not analyzed. Imaging was performed from sequential z-series scans with LSM5 PASCAL confocal microscope (Zeiss, Germany). For analysis of spines, three-dimensional images were constructed from ~40 sequential z-series sections of neurons scanned every 0.45 μm with a 63 × water immersion lens, NA 1.2 (Zeiss). The excitation and emission wavelengths were 488 and 515 nm, respectively. The applied zoom factor (3.0) yielded 23 pixels per 1 μm. The z-axis resolution was ~0.71 μm. The confocal lateral resolution was ~0.26 μm. Our resolution limits were regarded as sufficient to allow the determination of the density of thorns or spines.

Confocal images were then deconvoluted using AUTODEBLUR software (AutoQuant, USA). The density of spines as well as the head diameter was analyzed with Spiso-3D (a software calculating the center of spine and diameter of spine head based on the geometrical parameters of spines) developed by Kawato's group of Bioinformatics Project (Mukai et al., 2011). Spiso-3D has an equivalent capacity with Neurolucida (MicroBrightField, USA), furthermore, Spiso-3D considerably reduces human errors and experimenter labor. We analyzed the secondary dendrites in the stratum radiatum, lying between 100 and 250 μm from the soma. The spine density was calculated from the number of spines having a total length of 50–80 μm. Spine shapes were classified into three categories as follows. (1) A small-head spine whose head diameter is 0.2–0.4 μm. (2) A middle-head spine whose head diameter is 0.4–0.5 μm. (3) A large-head spine whose head diameter is 0.5–1.0 μm. These three categories were useful to distinguish complex differences of spines across the estrus cycle. Because the majority of spines (>95%) had a distinct head and neck, and stubby spines and filopodium did not contribute much to overall changes, we analyzed spines having a distinct head. While counting the spines in the reconstructed images, the position and verification of spines was aided by rotation of three-dimensional reconstructions and by observation of the images in consecutive single planes.

### Statistical analysis

Data are expressed as mean ± sem. For comparison of the level of hippocampus-synthesized steroids as well as analysis of spinogenesis, we used a One-Way ANOVA followed by Dunnett's test for multiple comparisons. A difference was considered significant at a value of ^*^p < 0.05 or ^**^p < 0.01.

## Results

### Determination of sex steroid levels in the female hippocampus

**Figure 1:**
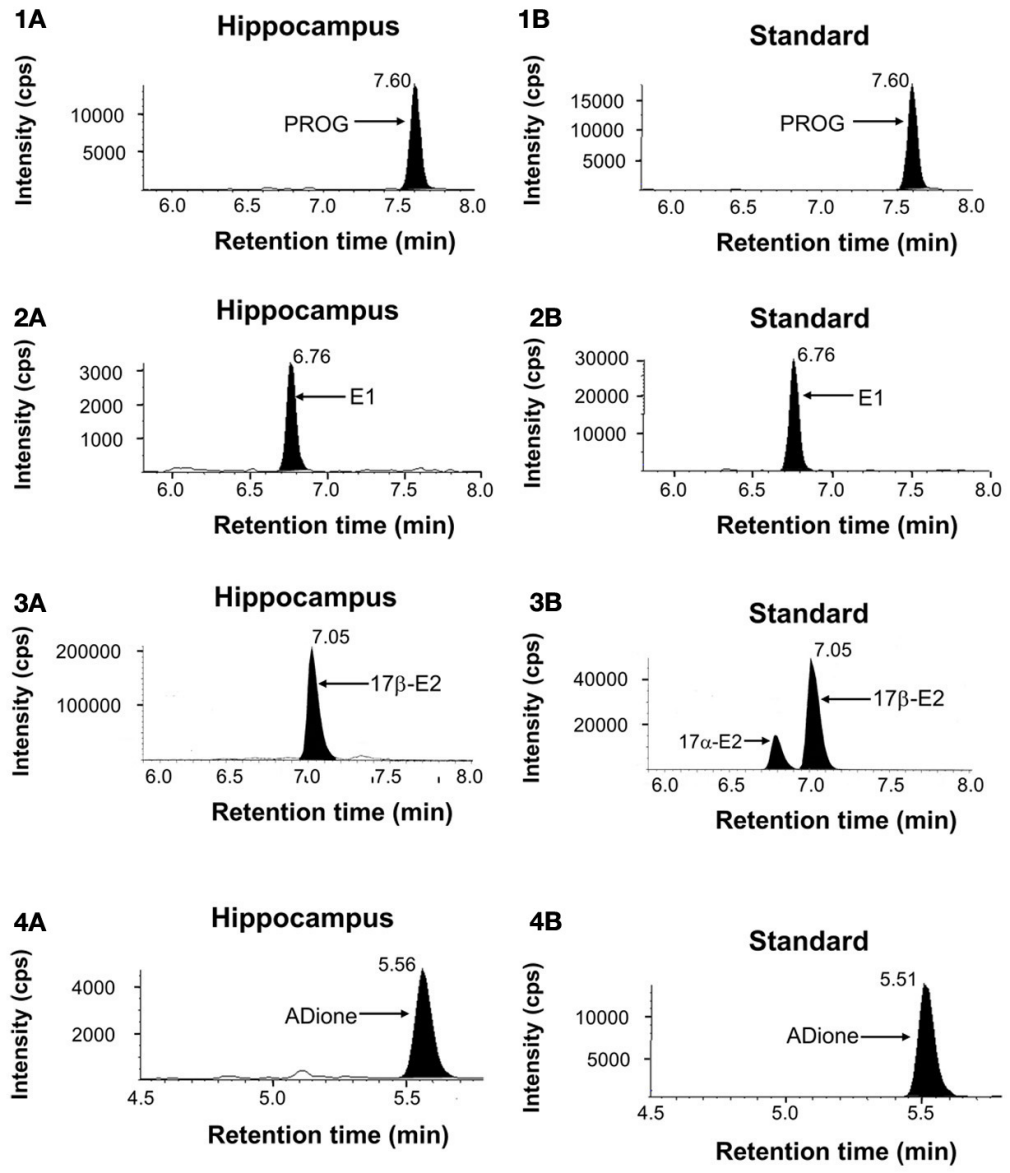
**Mass-spectrometric analysis of hippocampal sex-steroids (12-week-old rats).** LC-MS/MS ion chromatograms of PROG (1), E1 (2), E2 (3), and ADione (4). **(1A)**, **(2A)**, **(3A)**, and **(4A)** represent the chromatograms of the fragmented ions of each steroid from the hippocampus. Shaded portions indicate the intensity of fragmented ions of PROG (m/z = 97), E1 (m/z = 157), E2 (m/z = 339), and ADione (m/z = 109), respectively. **(1B)**, **(2B)**, **(3B)**, and **(4B)** represent the chromatograms of the fragmented ions of the standard steroids. The vertical axis indicates the intensity of the fragmented ion. The horizontal axis indicates the retention time of the fragmented ion, *t* = 7.60 min for PROG, 6.76 min for E1, 7.05 min for E2, and 5.56 min for ADione. The time of sample injection to LC system was defined as *t* = 0 min. Note that pre-purification step using normal phase HPLC before injection to LC system is very important to achieve high precision and good reproducibility of LC-MS/MS determination in order to avoid contamination of other steroids and fats. Steroid-derivatives or steroids were further separated with reversed phase LC-column before MS/MS. In the multiple reaction monitoring mode, the instrument monitored the m/z transition (Table S1).

**Figure 2:**
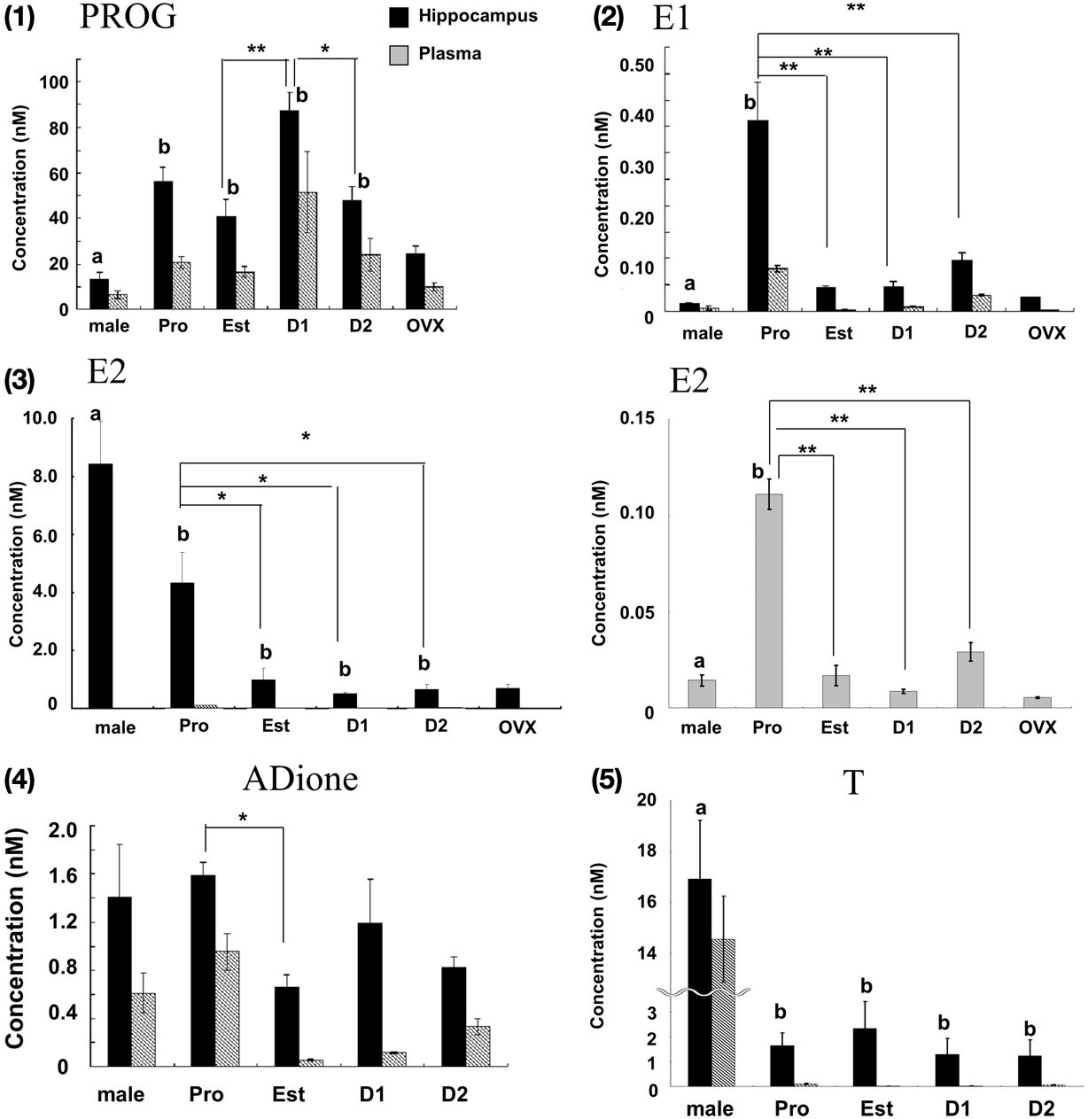
**Cyclic fluctuation of the levels of PROG (1), E1 (2), E2 (3), ADione (4), and T (5) in the female hippocampus and plasma.** In each panel, from left to right, male hippocampus (male), female hippocampus at Pro (P), Est (E), D1 (D1), D2 (D2), and OVX female hippocampus (OVX). For comparison, male data are taken from Hojo et al. (2009). In panel (3), the magnified graph for plasma is also displayed, because the level of E2 in plasma was much lower than that in the hippocampus. For each estrous cycle stage measurement, 3–6 rats were used, and totally 16 rats were used. Four OVX rats were also used. Each value is mean ± sem. Statistical significance between 4 stages (Pro, Est, D1 and D2) was calculated by One-Way ANOVA followed by Dunnett's *post hoc* comparison. Concerning the *post hoc* comparison, D1 for PROG and Pro for other steroids were used as control. ^*^p < 0.05; ^**^p < 0.01. Concerning the comparison between male and female, analysis was performed in same manner as above, except for male group as control. Statistical significance with *p* < 0.01 was observed between male and female which are indicated by “a” or “b.”

The concentration of sex steroids was determined for adult female rat hippocampus using a chromatogram analysis of the fragmented ions of steroid-derivatives. Results are summarized in Figures 1, 2.

E1, E2, and T need derivatization before application to LC-MS/MS to determine their accurate concentrations in the brain where the absolute content of steroids is extremely low. We employ picolinoyl derivatization in order to improve limit of quantification (LOQ) (Table S1). In case of E2, pentafluorobenzyl (PFBz)-derivatization was additionally performed simultaneously, in order to increase evaporation probability in electrospray ionization procedures.

**Figure 3:**
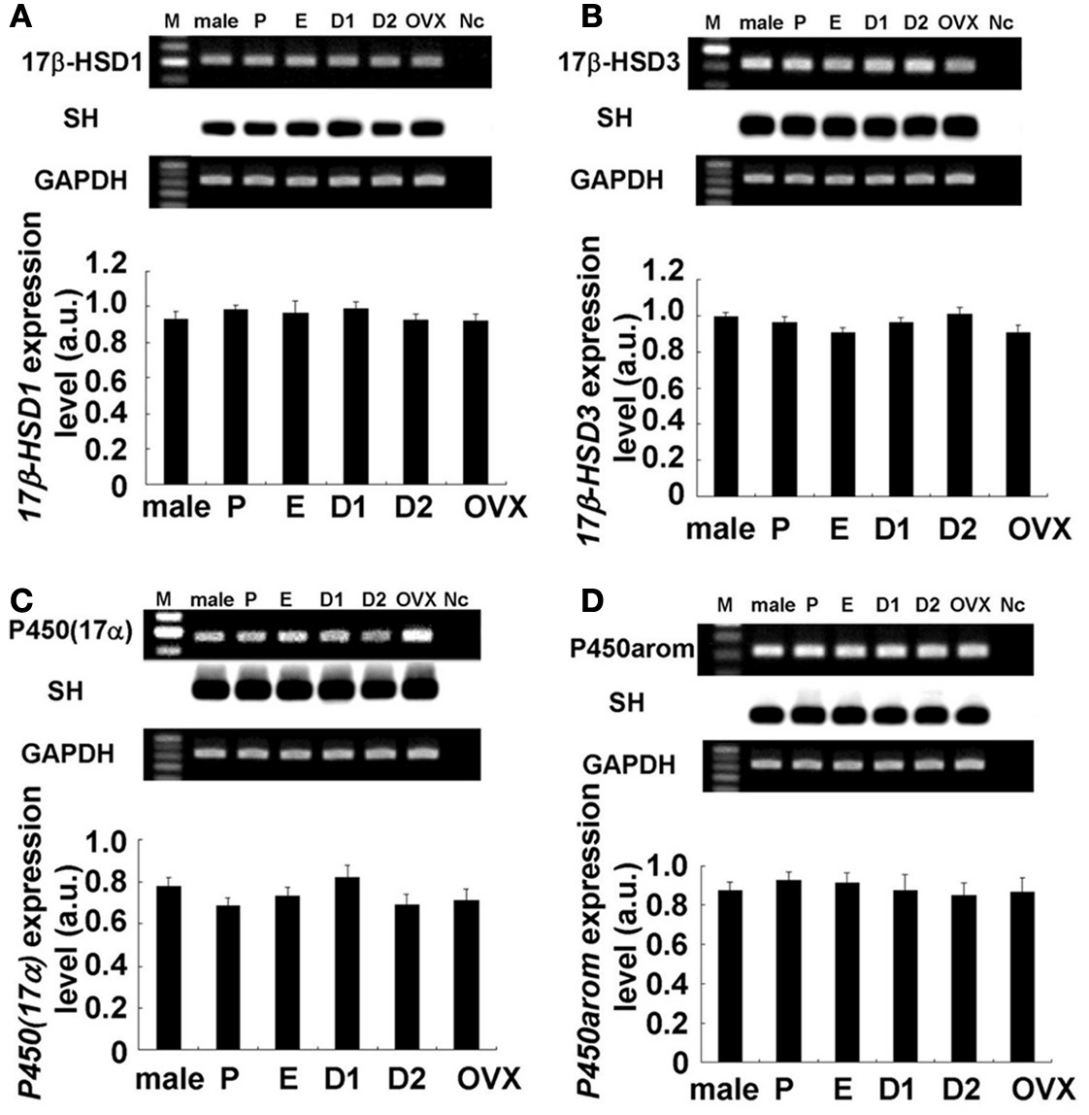
**No significant change in expression levels of mRNAs for sex steroidogenic enzymes [A: 17β-HSD1, B: 17β-HSD3, C: P450(17α), and D: P450arom] in the female hippocampus across the estrus cycle.** Upper panels show representative PCR images and lower panels show statistical comparisons. In each images, from left to right, size marker (100 bp ladder) (M), male hippocampus (male), female hippocampus at Pro (P), Est (E), D1 (D1), D2 (D2), and OVX female rats (OVX), the sample without template DNA as negative control (Nc). For each enzyme, the RT–PCR products for mRNAs are visualized with ethidium bromide staining on the top of each panel. Southern hybridization (SH) of cDNA is also shown on the middle of each panel. As an internal control, the ethidium bromide staining of GAPDH is shown on the bottom of each panel. PCR was performed by using cDNA made by reverse transcription from 100 ng of hippocampal total RNA. Statistical comparisons show no estrus cycle-dependent changes of mRNA expression for sex-steroidogenic enzymes. The vertical axis indicates the expression level for each enzyme calculated from the intensity of EB bands. Each value is mean ± sem. Data are taken from duplicate determinations for each rat of total 4 rats.

**Figure 4:**
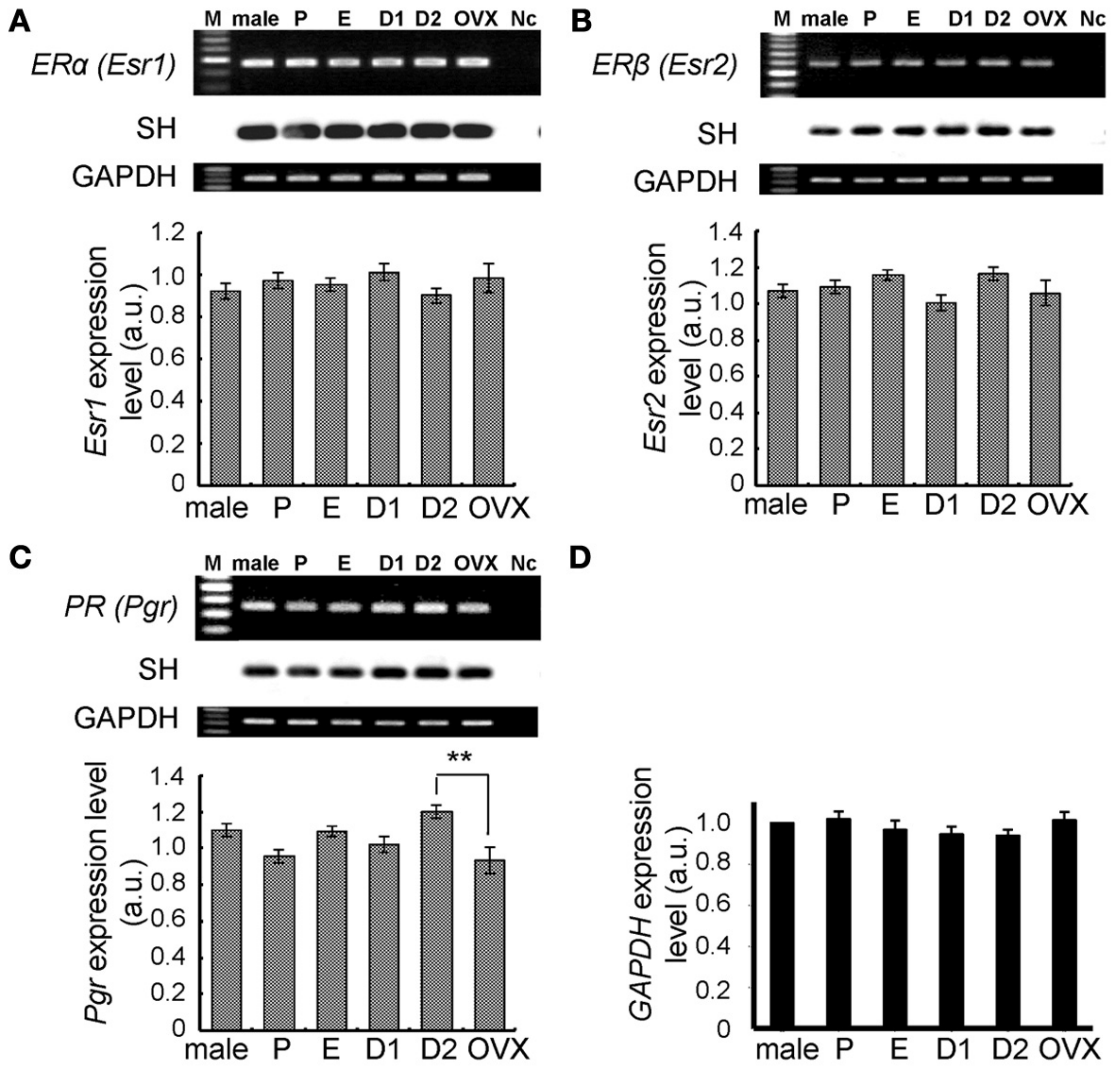
**No significant change in expression levels of mRNAs for steroid receptors (ERα, ERβ, and PR) and GAPDH in the female hippocampus across the estrus cycle.** Upper panels show representative PCR images and lower panels show statistical comparisons. In each images, from left to right, size marker (100 bp ladder) (M), male hippocampus (male), female hippocampus at P, E, D1, D2, and OVX female rats, the sample without template DNA as negative control (Nc). Ethidium staining and Southern hybridization (SH) are shown. PCR was performed by using cDNA made by reverse transcription from 100 ng of hippocampal total RNA. Statistical comparisons show very weak estrus cycle dependent changes of mRNA expression for steroid receptors **(A–C)** and GAPDH **(D)**. The vertical axis indicates the expression level for each enzyme calculated from the intensity of EB bands. For GAPDH, relative expression level against that in male (normalized to 1.0) is presented. Each values are mean ± sem. Statistical significance, ^*^p < 0.05; ^**^p < 0.01. Data are taken from duplicate determinations for each rat of total 4 rats.

In the chromatographic profiles of the fragmented ion of PROG and E1-3-picolinoyl-ester, a single clear peak was observed at 7.60 and 6.76 min, respectively (Figures 1-1A, 1-2A). For these steroids, the retention time of the observed steroid peak was the same as that of standard steroid (Figures 1-1B, 1-2B). Chromatographic profiles for the fragmented ions of E2-PFBz-picolinoyl showed a clear peak with the retention time of 7.01 min which was the same as that of the standard E2 derivative (Figures 1–3). In the chromatographic profiles of the fragmented ion of ADione and T-17-picolinoyl-ester, a major peak was observed at 5.56 and 4.84 min, respectively (Figures 1–4).

To confirm the assay accuracy, the hippocampal homogenate spiked with known amounts of the steroids was prepared and its concentration of steroid was determined (Table S3). Satisfactory accuracy was obtained, supporting the accuracy of determined hippocampal steroid content in Figure 2 and Table 1. The LOQs were defined in Table S1 as the lowest value with an acceptable accuracy (90–110%) and precision (i.e., RSD <10%). The results of intra-and inter-assay were shown in Table S1. The RSD for intra-and inter-assay was less than 6.3 and 9.2%, respectively.

**Table 1:** **Mass spectrometric analysis of the concentration of steroids in the hippocampus and plasma of adult female rats**.

	**Proestrus**	**Estrus**	**Diestrus 1**	**Diestrus 2**	**OVX**
**(A) Hippocampus**					
PROG (nM)^a^	55.7 ± 6.8^b^ (*n*^c^ = 4)	40.7 ± 7.8 (*n* = 4)	87.0 ± 8.1 (*n* = 3)	48.0 ± 6.2 (*n* = 4)	24.5 ± 3.3 (*n* = 5)
PROG (ng/g wet weight)	17.5 ± 2.1	12.8 ± 2.5	27.4 ± 2.5	15.1 ± 1.9	7.7 ± 1.0
El (nM)	0.36 ± 0.07 (*n* = 4)	0.045 ± 0.004 (*n* = 4)	0.05 ± 0.0l (*n* = 4)	0.10 ± 0.01 (*n* = 4)	0.025 ± 0.003 (*n* = 3)
El (ng/g wet weight)	0.097 ± 0.019	0.012 ± 0.001	0.014 ± 0.003	0.027 ± 0.003	0.007 ± 0.001
17ß-E2 (nM)	4.3 ± 1.0 (*n* = 6)	1.0 ± 0.4 (*n* = 4)	0.51 ± 0.05 (*n* = 3)	0.7 ± 0.1 (*n* = 4)	0.7 ± 0.1 (*n* = 4)
17ß-E2 (ng/g wet weight)	1.18 ± 0.27	0.27 ± 0.11	0.14 ± 0.01	0.33 ± 0.03	0.33 ± 0.03
ADione (nM)	1.6 ± 0.1 (*n* = 4)	0.7 ± 0.1 (*n* = 4)	1.1 ± 0.4 (*n* = 4)	0.83 ± 0.08 (*n* = 4)	
ADione (ng/g wet weight)	0.46 ± 0.03	0.20 ± 0.03	0.32 ± 0.11	0.24 ± 0.02	
T (nM)	1.6 ± 0.5 (*n* = 4)	2.3 ± 1.1 (*n* = 4)	1.3 ± 0.6 (*n* = 3)	1.2 ± 0.7 (*n* = 4)	
T (ng/g wet weight	0.46 ± 0.14	0.66 ± 0.32	0.37 ± 0.17	0.35 ± 0.20	
**(B) Plasma**					
PROG (nM)	20.5 ± 2.6 (*n* = 4)	16.7 ± 2.3 (*n* = 4)	51.6 ± 17.9 (*n* = 3)	24.1 ± 7.3 (*n* = 4)	10.1 ± 1.6 (*n* = 5)
PROG (ng/g wet weight)	4.4 ± 0.8	5.3 ± 0.7	16.2 ± 5.6	7.6 ± 2.3	3.2 ± 0.5
E1 (nM)	0.082 ± 0.006 (*n* = 4)	0.004 ± 0.001 (*n* = 4)	0.009 ± 0.001 (*n* = 4)	0.031 ± 0.002 (*n* = 4)	0.002 ± 0.001 (*n* = 4)
E1 (ng/g wet weight)	0.022 ± 0.002	0.001 ± 0.000	0.002 ± 0.000	0.008 ± 0.001	0.001 ± 0.000
17ß-E2 (nM)	0.111 ± 0.008 (*n* = 6)	0.017 ± 0.005 (*n* = 6)	0.009 ± 0.001 (*n* = 5)	0.029 ± 0.005 (*n* = 6)	0.005 ± 0.000 (*n* = 5)
17ß-E2 (ng/g wet weight)	0.030 ± 0.002	0.005 ± 0.001	0.002 ± 0.000	0.008 ± 0.001	0.001 ± 0.000
ADione (nM)	1.0 ± 0.2 (*n* = 4)	0.06 ± 0.01 (*n* = 4)	0.119 ± 0.005 (*n* = 4)	0.33 ± 0.07 (*n* = 4)	
ADione (ng/g wet weight)	0.29 ± 0.06	0.017 ± 0.003	0.034 ± 0.001	0.06 ± 0.02	
T (nM)	0.10 ± 0.03 (*n* = 4)	0.013 ± 0.004 (*n* = 4)	0.020 ± 0.007 (*n* = 3)	0.06 ± 0.02 (*n* = 4)	
T (ng/g wet weight)	0.029 ± 0.009	0.004 ± 0.001	0.006 ± 0.002	0.017 ± 0.006	
**(C) OVX ± E2^d^**					
		**Hippocampus**		**Plasma**	
17ß-E2 (nM)		3.8 ± 0.4 (*n* = 3)		1.3 ± 0.4 (*n* = 4)	
17ß-E2 (ng/g wet weight)		1.0 ± 0.1		0.36 ± 0.10	

a Concentration in nM is calculated using the average volume of 0.124 mL for one whole hippocampus that has 0.124 ± 0.002 g wet weight (n = 44). We assumed that tissue having 1 g of wet weight has an approximate volume of 1 ml, since the major part of tissue consists of water whose 1 mL weight is 1 g. The volume should be decreased by less than 10%, due to the specific volumes of proteins and lipids (0.7–0.8 ml/g) (see McIlwain, 1985). Intact shows the averaged values from intact and sham-operated rats, because there were no significant differences between these two groups of rats.

b Data are expressed as mean ± sem.

c Number of animals (i.e., the number of hippocampi).

d E2 (40 μg/kg body weight) was s.c. injected 5 h before measurements.

In order to compare hippocampal levels of steroids with plasma steroids, we converted ng/g wet weight to nM concentration *via* the following estimation (see Table 1). First, 1 mL of Plasma (93% is water) is assumed to have 1 g weight, as 1 mL of water has 1 g weight. Second, we assume that the hippocampal tissue having 1 g of wet weight has an approx. volume of 1 mL, as nearly 78% of the brain tissue consists of water (McIlwain, 1985). Consideration of specific volume of protein and lipids (0.7–1.0 mL/g) in the brain further support this assumption (Tanford et al., 1974; Xie and Stone, 1986; Kimoto et al., 2001).

Using the average hippocampal volume of 0.124 mL (deduced from 0.124 ± 0.002 wet weight for one whole hippocampus of 12 weeks old female rat, *n* = 44), the concentrations of E2, T, PROG, ADione and E1 in the hippocampus were calculated at each stage of estrous cycle. Based on these considerations, 0.95 ng/g wet weight of E2 in the hippocampus at Pro corresponds to 4.3 nM. (Table 1).

### Cyclic fluctuation of female sex steroids (PROG, E1, and E2)

#### PROG (Figure 2-1; Table 1)

The hippocampal PROG showed cyclic fluctuation, having good correlation with the plasma estrous cycle, with a peak at D1 (~87 nM) and low level at Pro, Est, and D2 (40–50 nM). The level of hippocampal PROG was approximately 2-fold of that of circulating PROG along the entire estrous cycle. If we assume that all the circulating PROG can penetrate into the hippocampus, a possible contribution of hippocampus-synthesized PROG may be 25–35 nM, approximately a half of the total PROG in the hippocampus, independent of the estrous cycle. Female hippocampal level of PROG was higher than male one at all stages of estrous cycle.

#### E1 (Figure 2-2; Table 1)

The hippocampal E1 showed cyclic fluctuation, correlated with the plasma estrous cycle, with a peak at Pro (~0.36 nM) and low level at Est-D2 (0.05–0.07 nM). The phase of E1 cycle in the hippocampus looked similar to that in plasma. However, the E1 level was much higher (4–8 fold higher) in the hippocampus than that in plasma, indicating the net synthesis of E1 in the hippocampus. Interestingly, female hippocampal E1 was much higher than male (~0.015 nM) (Hojo et al., 2009).

#### E2 (Figure 2-3; Table 1)

The level of E2 in the female hippocampus showed a peak at Pro (~4.3 nM), and decreased at Est, D1 and D2 (1.0–0.5 nM). Hippocampal E2 level was approximately 10–60-fold higher than that in plasma, suggesting that almost all the hippocampal E2 is synthesized in the hippocampus. Interestingly, at all stages of estrous cycle, E2 level in the female hippocampus was much lower than that in male (~8.4 nM) (Hojo et al., 2009).

The level of PROG, E1, and E2 in the female hippocampus was relatively high in nanomolar range, although the absolute content of E2 was only ~0.11 ng (at Pro) in one hippocampus, because of the small volume of one hippocampus (~0.12 mL).

### Effect of OVX on the hippocampal sex steroid levels

By using the OVX rats, we investigated the net hippocampal synthesis level of sex steroids under the depletion of circulation-derived sex steroids, since OVX procedures eliminate the contribution of sex steroids via blood circulation.

Upon OVX, the level of hippocampal sex steroids reached nearly its minimal level over 4 stages in the estrous cycle, but did not become zero.

By OVX, plasma E2 was completely depleted (~0.005 nM), whereas the hippocampal E2 decreased to ~0.7 nM, which was identical to the level at Diestrus. OVX also decreased the hippocampal PROG to ~24.5 nM, close to the level at Est. A significant level of hippocampal E1 was remained (~0.025 nM) even after OVX, although the circulation-derived E1 was depleted (~0.002 nM). This ~25 pM of E1 was close to the level at Est stage. These results imply that the synthesis activity of E2, PROG, and E1 in the female hippocampus is significant. The remaining PROG (~10.1nM) in plasma even after OVX may be derived from the adrenal gland via circulation.

E2 replacement effects on OVX rats were investigated. E2 (40 μg/kg body weight) was subcutaneously injected into OVX rats 5 h before measurements. Upon s.c. injection, hippocampal E2 level elevated to 3.8 nM that was almost identical to hippocampal E2 level at Pro, whereas plasma E2 level was 1.3 nM that was much higher than plasma E2 level at Pro (Table 1C).

### Androgens (adione and T) in the female hippocampus

**Figure 5:**
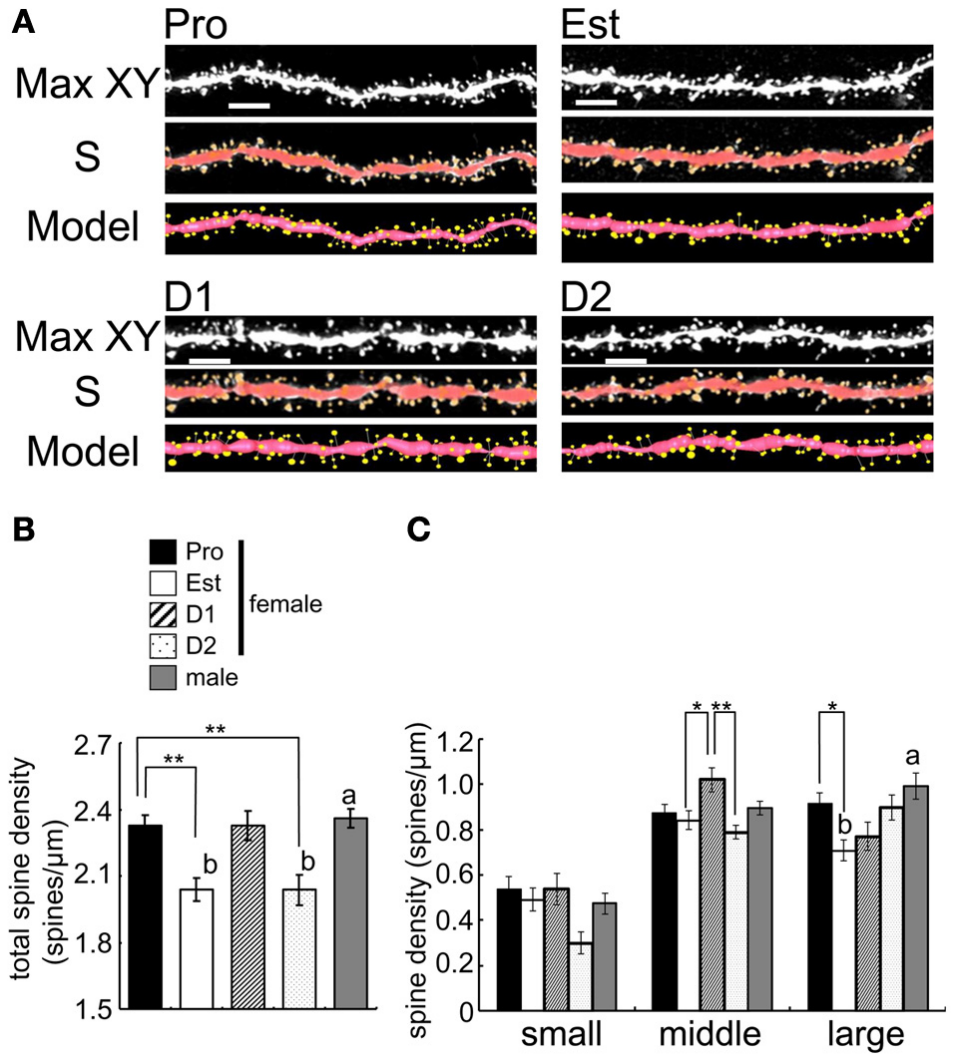
**Cyclic fluctuation of the density and morphology of spines across the estrous cycle in CA1 region of the female hippocampus (perfusion-fixed slices). (A)** Spine images along the secondary dendrites in the stratum radiatum. Maximal intensity projections onto XY plane from z-series confocal micrographs (Max XY) for Pro, Est, D1, and D2. Spine images analyzed by Spiso-3D (S) and 3 dimensional model illustration (Model). **(B)** Total spine density of CA1 neurons of female and male hippocampus. From left to right, Pro (black column), Est (white column), D1 (hatched column), D2 (dotted column), and male (gray column). **(C)** Density of three subtypes of spines, i.e., small-head spines (small), middle-head spines (middle), and large-head spines (large). Male spine data are nearly the same as those of female at Pro. Vertical axis is the average number of spines per 1 μm of dendrite. For each estrus stage of female or male, we investigated 3 rats, 6 slices, 12 neurons, 24 dendrites, and 3000–4000 spines. Female Pro is used for control. Statistical significance between 4 stages was calculated by One-Way ANOVA followed by Dunnett's *post-hoc* comparison. ^*^p < 0.05; ^**^p < 0.01. Statistical significance with *p* < 0.01 was observed between male and female which are indicated by “a” or “b.” Scale bar, 5 μm.

In addition to hippocampal female sex steroids, we also determined the level of androgens (ADione and T) in the female hippocampus. Determination of hippocampal androgens is important, because ADione and T are the precursors for E1 and E2, respectively. The hippocampal level of ADione and T at each stage of estrous cycle was much higher than that in plasma, suggesting the hippocampal androgen synthesis is significant in the female hippocampus (Figures 2-4, 2-5).

The hippocampal level of ADione in the female changed along the estrous cycle, but peaked at both Pro and D1, whereas plasma ADione showed a peak only at Pro.

The hippocampal level of T did not considerably change along the estrous cycle (1.2-2.3 nM) (Figures 2–5, Table 1). On the other hand, plasma T clearly showed a peak at Pro (~0.10 nM). Hippocampal T was much higher than plasma T, indicating the hippocampal synthesis of T.

### Sex differences of the hippocampal level of sex hormones

The levels of PROG and E1 in the female hippocampus were 3-10 times higher at all the stages of estrous cycle than those in male (Hojo et al., 2009). On the other hand, E2 level in the female hippocampus was much lower (~1/8) than that in male except at Pro. The level of T in the female hippocampus was much lower (~1/10) than that in male.

It should be noted that the levels of plasma estrogens and androgens in Figure 2 showed typical estrous cycle dependent changes as previously reported elsewhere (Watanabe et al., 1990).

### Molecular biological analysis of steroidogenic enzymes and steroid receptors in the female hippocampus

The hippocampal expression of steroidogenic enzymes [P450arom, 17β-HSD (types 1 and 3) and P450(17α)] were examined. Typical RT-PCR images and comparison of mRNA expression levels were shown in Figures 3, 4. Surprisingly, no significant cyclic fluctuations across the estrus cycle were observed for their expression levels (normalized by GAPDH expression). Note that GAPDH expression itself was not changed across the estrus cycle. OVX did not change the expression level of any steroidogenic enzyme. Relative number of transcripts, expressed in the hippocampus of adult female rats, was ~1/600 of that in the ovary for P450arom, ~1/200 of that in the ovary for 17β-HSD (type 1), ~1/300 of that in the testis for 17β-HSD (type 2), and ~1/300 of that in the testis for P450(17α).

Again, no significant cyclic fluctuations across the estrus cycle were observed for the expression level of hippocampal ERα and ERβ (Figure 4). The expression level of PROG receptor (PR) was also not significantly changed, although a slight elevation at D2 was observed. Relative number of transcripts, expressed in the hippocampus of adult female rats, was ~1/30 for ERα, ~1/80 for ERβ, and ~1/10 for PR, respectively, of the ovary.

### Cyclic fluctuation of spine density in the female hippocampus

We examined the spine density and morphology of spines in four stages of estrous cycle in CA1 hippocampal neurons with perfusion-fixed slices. A significant cyclic fluctuation of spine density across the estrus cycle occurred (Figure 5). The total spine density showed the maximum at Pro, decreased from Pro to Est, increased from Est to D1, decreased from D1 to D2, and increased from D2 to Pro. Further detailed analysis of head diameter distribution allowed us to distinguish Pro spines from D1 spines, although they had almost the same density. At Pro, large-head spines (0.5–1.0 μm) are dominant whereas middle-head spines (0.4–0.5 μm) are dominant at D1.

### PROG can increase the spine density as examined in acute slices (Figure 6)

We tried to examine the effects of E2 and PROG on the spine density by using acute male hippocampal slices whose sex steroids were depleted (below 0.5 nM) due to steroid release to artificial cerebrospinal fluid during recovery incubation (Hojo et al., 2011; Ooishi et al., 2012). The spine density was significantly increased by the incubation of slices with 100 nM PROG which mimicked the PROG level at D1 (~90 nM PROG).

## Discussion

### Female hippocampal levels of sex steroids are higher than circulation levels

Until the current study, hippocampal levels of sex steroids (PROG, E1, and E2) at each stage of the estrous cycle had not been demonstrated in female animals, although the estrous cycle-dependent fluctuation of circulating sex steroids is well known (Watanabe et al., 1990). The hippocampal levels of sex steroids were much higher than those in plasma, implying the net hippocampal synthesis of sex steroids does occur (Figure 2).

### Cyclic fluctuation of hippocampal E2, E1, and PROG

The cyclic changes of E1, E2, and PROG in the hippocampus were observed. E1 and E2 showed a peak at Pro and PROG showed a peak at D1. We can interpret these phenomena as follows. The mRNA expression levels of the enzymes for estrogen synthesis did not change significantly along the estrous cycle (Figure 3). Therefore, the capacity of endogenous synthesis of hippocampal estrogens (PROG, E1, and E2) may approx. be the same between all the 4 stages of the estrous cycle. In this case, hippocampus-synthesized PROG (~30 nM) may almost be the same between all the 4 stages, therefore the addition of fluctuating plasma PROG (20–50 nM with a peak at D1, penetrating to the hippocampus) can create the cyclic fluctuation of the total hippocampal PROG which was synchronized with the plasma estrous cycle (Figure 2-1). Here, circulating PROG can penetrate into the hippocampus (Pardridge and Mietus, 1979). Fluctuating plasma ADione and E1 (with a peak at Pro) may also contribute to fluctuation of hippocampal ADione and E1. As a result, the cyclic fluctuation of hippocampal E2 across the estrous cycle may be resulted from the conversion of fluctuating PROG/ADione/E1 to E2 by hippocampal steroidogenic systems.

Since the hippocampal E2 is much higher (10–60 fold for E2) than that in plasma, a penetration of the circulating E2 into the hippocampus, however, cannot account for this cyclic fluctuation of hippocampal E2. The phase of fluctuation of hippocampal E2 is not coincident with the phase of PROG fluctuation, which may be due to the phase shift along several steps of steroid conversion from PROG to E2.

By using OVX, we can evaluate possible fluctuation of hippocampus-derived steroidogenic capacity. In five OVX rats (plasma PROG fluctuation is depleted), we observed very small variation in hippocampal PROG, E1, and E2 (Figure 2), implying that hippocampal cyclic fluctuation should mainly be due to plasma PROG/ADione fluctuation. Between five OVX rats, we also observed no variation in hippocampal E2 and plasma E2. These findings imply that fluctuation of hippocampal E2 is mainly caused by plasma PROG/ADione fluctuation. Therefore, cyclic steroidogenic capacity of ovary probably adds fluctuation to hippocampal steady-state steroidogenic capacity that is deduced from the steady-state mRNA expression across the estrus cycle.

The expression level of ERα, ERβ, and PR did not change significantly across the estrous cycle. These results also suggest that the cyclic change in the spine density is mainly induced by the cyclic fluctuation of PROG and E2 in the hippocampus, but not by fluctuation of receptors.

### Earlier studies for OVX and spine change

Historically, it had been believed that the depletion of the circulation-derived E2 can cause the considerable decrease in E2 in the brain, because of the belief that the brain does not synthesize E2 (Woolley and McEwen, 1993; MacLusky et al., 2005; Chen et al., 2009). Figure 2 shows that upon OVX, the level of hippocampal estrogens reached nearly its minimal level across 4 stages in the estrous cycle (i.e., D1), still much higher than the highest plasma E2 level at Pro (~0.1 nM). OVX did not change the expression level of any steroidogenic enzymes (Figure 3). These results suggest that the female hippocampus keeps a significant activity of estrogen synthesis even after the depletion of circulation-derived estrogens.

The density of spines decreases by 25–50% after OVX in hippocampal CA1 neurons (Gould et al., 1990; Woolley and McEwen, 1993). These observations have been explained as the results from depletion of plasma E2. This decrease in the density of spines can be partially explained by the decrease in the hippocampal E2 level to ~0.7 nM (similar to D1 and D2).

**Figure 6:**
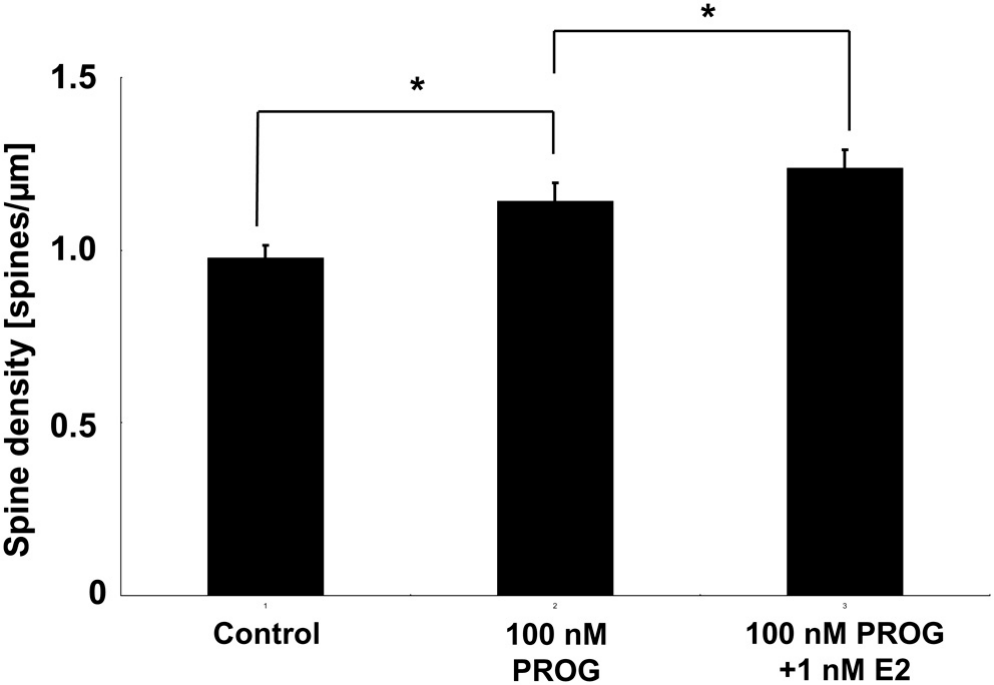
**Effect of E2 and PROG on spines of CA1 neurons in acute slices of the hippocampus.** Vertical axis is the total number of spines per 1 μm dendritic segment. Results are represented as means ± sem. ^*^p < 0.05. For each drug treatment, we investigated 3 rats, 6 slices, 12 neurons, 24 dendrites, and 1400–2000 spines.

Another factor of spine decrease may be a decline in hippocampal PROG. Upon OVX, hippocampal PROG decreased to the level which is lower than the lowest level of PROG across the estrous cycle (Est). Both PROG and E2 showed enhancement effects on the spinogenesis (Figure 6; (Mukai et al., 2007)). Taken together, the effect of E2 and PROG on spinogenesis can be explained by their hippocampal level rather than plasma level.

As a model of E2 replacement therapy, the decrease in the density of spines in OVX rats is rescued by s.c. injection of E2 (Woolley and McEwen, 1993; MacLusky et al., 2005). These results can be explained by increase in the level of hippocampal E2 by penetration of injected E2. To verify the explanation, we measured hippocampal E2 upon E2 replacement. Subcutaneous injection of E2 (40 μg/kg body weight) elevated the hippocampal E2 level to 3.8 nM that was almost identical to that at Pro (Table 1C), supporting that such a low dose of E2 injection can be powerful enough to rescue the spine density of OVX rats in early studies (Woolley and McEwen, 1993; MacLusky et al., 2005).

### Cyclic fluctuation of hippocampal spine density due to cyclic fluctuation of total hippocampal PROG and E2

We newly found out the difference in dendritic spine density between at D1 and D2. The spine density showed the maximum at Pro, decreased from Pro to Est, increased to D1 (almost same level as Pro), decreased to D2 (almost same level as Est), and increased again to Pro (Figure 5). If spine density at D1 and D2 are averaged as Diestrus (D), the spine density at D is an intermediate level between Pro and Est, which is consistent with the previous study (Woolley et al., 1990). Importantly, earlier studies did not distinguish D1 from D2 concerning the spine density and morphology as well as E2/PROG levels.

In order to explain the observed cyclic fluctuation of the spine density, we consider the contribution of not only E2 level but also PROG level. Hippocampal level of E2 alone does not account for it because hippocampal E2 at D1 is the lowest (0.5 nM) whereas the spine density at D1 is the highest. The effect of PROG should be considered. At D1, hippocampal PROG with the peak value of 90 nM may significantly increase the spine density, while 0.5 nM E2 may be less effective on spinogenesis (Mukai et al., 2007). At Pro, in contrast, hippocampal E2 (4.3 nM on average) may significantly contribute to the spinogenesis, because hippocampal PROG level at Pro is approx. half of D1. This hypothesis is supported by the experiments on spinogenesis using isolated acute hippocampal slices. As shown in Figure 6, 100 nM of PROG (close to D1 condition) significantly elevated spine density.

### Consideration of kinase activity or synaptic protein expression

The kinase activity, steroidogenic enzyme activity or synaptic protein expression levels may fluctuate across the estrous cycle, although the mRNA expression levels of the enzymes for estrogen synthesis did not change significantly along the estrous cycle. For example, the order of phosphorylation level is Pro > Diestrus (D) (D1 plus D2) for Erk MAP kinase, Pro > D > Est for Akt kinase, and Pro > Est ~ D for LIM kinase (LIMK), respectively (Bi et al., 2001; Znamensky et al., 2003; Spencer et al., 2008). Note that the activity of P450arom (E1/E2 synthase) decreases upon phosphorylation by PKA or PKC (Balthazart et al., 2006). If the activity of PKA or PKC fluctuates, then following P450arom activity change may generate hippocampal E1/E2 fluctuation across the estrous cycle. Note that in these studies, D1 and D2 are mixed up as Diestrus (D).

E2 may play a key role in the regulation of the phosphorylation level of kinases and synaptic proteins. E2 replacement in OVX rats increases the phosphorylation of Erk MAPK and NR2 subunit of NMDA receptors in the hippocampus (Bi et al., 2001). The expression level of PSD-95 is higher at Pro than Est or D (Znamensky et al., 2003; Spencer et al., 2008; Yildirim et al., 2008). Activation of MAPK is essential for E2-induced spinogenesis (Mukai et al., 2007, 2010).

Therefore, the fluctuation of hippocampus-synthesized sex steroids (E2, E1, and PROG) might also change in hippocampal spinogenesis (Figure 5) (Woolley and McEwen, 1992) or magnitude of LTP (Warren et al., 1995; Good et al., 1999; Bi et al., 2001) via change in the phosphorylation level of kinases or expression levels of synaptic proteins.

### Difference between female and male in the hippocampal synthesis of steroids

Surprisingly, the expression levels of sex steroidogenic enzymes including P450arom, 17β-HSD and P450(17α) were not significantly different between female and male hippocampus (Figure 3).

On the other hand, the level of sex-steroids (such as PROG, T, and E2) is significantly different between female and male. It may be due to the difference in precursor steroids supplied via circulation between female and male. Most abundant precursor steroids for synthesis of sex steroids in the hippocampus may be PROG from ovary (20–50 nM) in female, and T from testis (~15 nM) in male, respectively. The level of PROG in the female hippocampus was 3–6 fold higher than that in male over all the 4 stages of the estrous cycle. The level of T in the female hippocampus was much lower (~1/10) than that in male. Interestingly, E2 level in the female hippocampus (0.6–4.3 nM) was lower than that in male (~8 nM). The high level of male hippocampal E2 (8 nM) may be, partly due to, conversion from circulating (high level) T (~15 nM), penetrating into the hippocampus. The moderately high level of female hippocampal E2 (0.5–4.3 nM) may be mainly due to conversion from circulating PROG (20–50 nM) in addition to hippocampus-synthesized PROG, since circulating E2, E1, T were too low (<1 nM) to contribute.

### Conflict of interest statement

The authors declare that the research was conducted in the absence of any commercial or financial relationships that could be construed as a potential conflict of interest.

## Supplementary material

Figure S1 **Steroid derivatives and their fragmented ions used for analysis with LC-MS/MS. (A)** PROG (m/z = 315) and its fragmented ion (m/z = 97), **(B)** E1-3-picolinoyl-ester (m/z = 376) and its fragmented ion (m/z = 157), **(C)** E2-3-pentafluorobenzoxy-17-picolinoyl-ester (m/z = 558) and its fragmented ion (m/z = 339), **(D)** ADione (m/z = 287) and its fragmented ion (m/z = 109), **(E)** T-17-picolinoyl-ester (m/z = 394) and its fragmented ion (m/z = 253). Picolinoyl-ester is used for induced-inonization, and pentafluorobenzoxy-ester is particularly used to increase evaporation probability of E2. Note that PROG and ADione were measured without derivatization.

Figure S2 **Calibration curves for LC-MS/MS using standard steroids dissolved in ethanol.** Horizontal (x) axis indicates the concentration of added standard steroid. Vertical (y) axis indicates the relative intensity obtained from the chromatogram. **(A)** Calibration curve for PROG. Linearity was observed between 2 pg/mL and 1250 pg/mL. **(B)** Calibration curve for E1. Linearity was observed between 1 pg/mL and 1000 pg/mL. **(C)** Calibration curve for E2. Linearity was observed between 0.1 and 1000 pg/mL. **(D)** Calibration curve for ADione. Linearity was observed between 2 and 1250 pg/mL. **(E)** Calibration curve for T. Linearity was observed between 0.5 and 1000 pg/mL.

Figure S3 **Amplification curves for steroidogenic enzymes and receptors.** The PCR cycle numbers within linear phase are indicated in the parentheses. Each PCR amplification curve was obtained from cDNA made by reverse-transcription from 100 ng of hippocampal total RNA from the hippocampus. These cDNA templates were used for the experiments shown in Figures 3, 4. See also Table S2.

Table S1 **The intra-and inter-assay of accuracy and precision as well as the limit of quantification (LOQ) for each steroid**.

Table S2 **The sequence of primer oligonucleotides for PCR amplification**.

Table S3 **The accuracy of steroid determination for hippocampal tissue spiked with exogenous steroids**.

## Abbreviations

ADione: androstenedione
D1: Diestrus 1
D2: Diestrus 2
E2: estradiol
E1: estrone
Est: Estrus
LC-MS/MS: liquid chromatography with tandem-mass-spectrometry
Pro: Proestrus
PROG: Progesterone
PFBz: pentafluorobenzoxy
T: testosterone.

## References

[B1] Balthazart J. Cornil C. A. Taziaux M. Charlier T. D. Baillien M. Ball G. F. (2006). Rapid changes in production and behavioral action of estrogens. Neuroscience 138, 783–791 10.1016/j.neuroscience.2005.06.016 16359807

[B2] Bi R. Foy M. R. Vouimba R. M. Thompson R. F. Baudry M. (2001). Cyclic changes in estradiol regulate synaptic plasticity through the MAP kinase pathway. Proc. Natl. Acad. Sci. U.S.A. 98, 13391–13395 10.1073/pnas.241507698 11687663 PMC60881

[B3] Chen J. R. Yan Y. T. Wang T. J. Chen L. J. Wang Y. J. Tseng G. F. (2009). Gonadal hormones modulate the dendritic spine densities of primary cortical pyramidal neurons in adult female rat. Cereb. Cortex 19, 2719–2727 10.1093/cercor/bhp048 19293395

[B4] Davis E. C. Popper P. Gorski R. A. (1996). The role of apoptosis in sexual differentiation of the rat sexually dimorphic nucleus of the preoptic area. Brain Res. 734, 10–18 10.1016/0006-8993(96)00298-3 8896803

[B6] Duan H. Wearne S. L. Morrison J. H. Hof P. R. (2002). Quantitative analysis of the dendritic morphology of corticocortical projection neurons in the macaque monkey association cortex. Neuroscience 114, 349–359 10.1016/S0306-4522(02)00305-6 12204204

[B7] Good M. Day M. Muir J. L. (1999). Cyclical changes in endogenous levels of oestrogen modulate the induction of LTD and LTP in the hippocampal CA1 region. Eur. J. Neurosci. 11, 4476–4480 10.1046/j.1460-9568.1999.00920.x 10594677

[B8] Gorski R. A. Gordon J. H. Shryne J. E. Southam A. M. (1978). Evidence for a morphological sex difference within the medial preoptic area of the rat brain. Brain Res. 148, 333–346 10.1016/0006-8993(78)90723-0 656937

[B9] Gorski R. A. Mennin S. P. Kubo K. (1975). The neural and hormonal bases of the reproductive cycle of the rat. Adv. Exp. Med. Biol. 54, 115–153 10.1007/978-1-4684-8715-2_6 1092141

[B10] Gould E. Woolley C. S. Frankfurt M. McEwen B. S. (1990). Gonadal steroids regulate dendritic spine density in hippocampal pyramidal cells in adulthood. J. Neurosci. 10, 1286–1291 2329377 10.1523/JNEUROSCI.10-04-01286.1990 PMC6570209

[B11] Hajszan T. MacLusky N. J. Johansen J. A. Jordan C. L. Leranth C. (2007). Effects of androgens and estradiol on spine synapse formation in the prefrontal cortex of normal and testicular feminization mutant male rats. Endocrinology 148, 1963–1967 10.1210/en.2006-1626 17317772 PMC2128740

[B12] HaoJ J. Janssen W. G. Tang Y. Roberts J. A. McKay H. Lasley B. (2003). Estrogen increases the number of spinophilin-immunoreactive spines in the hippocampus of young and aged female rhesus monkeys. J. Comp. Neurol. 465, 540–550 10.1002/cne.10837 12975814

[B13] Hojo Y. Hattori T. A. Enami T. Furukawa A. Suzuki K. Ishii H. T. (2004). Adult male rat hippocampus synthesizes estradiol from pregnenolone by cytochromes P45017alpha and P450 aromatase localized in neurons. Proc. Natl. Acad. Sci. U.S.A. 101, 865–870 10.1073/pnas.2630225100 14694190 PMC321772

[B14] Hojo Y. Higo S. Ishii H. Ooishi Y. Mukai H. Murakami G. (2009). Comparison between hippocampus-synthesized and circulation-derived sex steroids in the hippocampus. Endocrinology 150, 5106–5112 10.1210/en.2009-0305 19589866

[B15] Hojo Y. Higo S. Kawato S. Hatanaka Y. Ooishi Y. Murakami G. (2011). Hippocampal synthesis of sex steroids and corticosteroids: essential for modulation of synaptic plasticity. Front. Endocrinol. 2:43 10.3389/fendo.2011.00043 22701110 PMC3356120

[B16] Hojo Y. Murakami G. Mukai H. Higo S. Hatanaka Y. Ogiue-Ikeda M. (2008). Estrogen synthesis in the brain–role in synaptic plasticity and memory. Mol. Cell. Endocrinol. 290, 31–43 10.1016/j.mce.2008.04.017 18541362

[B17] Kawato S. Hojo Y. Kimoto T. (2002). Histological and metabolism analysis of P450 expression in the brain. Methods Enzymol. 357, 241–249 10.1016/S0076-6879(02)57682-5 12424914

[B18] Kimoto T. Ishii H. Higo S. Hojo Y. Kawato S. (2010). Semicomprehensive analysis of the postnatal age-related changes in the mRNA expression of sex steroidogenic enzymes and sex steroid receptors in the male rat hippocampus. Endocrinology 151, 5795–5806 10.1210/en.2010-0581 21047951

[B19] Kimoto T. Tsurugizawa T. Ohta Y. Makino J. Tamura H. Hojo Y. (2001). Neurosteroid synthesis by cytochrome p450-containing systems localized in the rat brain hippocampal neurons: N-methyl-D-aspartate and calcium-dependent synthesis. Endocrinology 142, 3578–3589 10.1210/en.142.8.3578 11459806

[B20] Komatsuzaki Y. Hatanaka Y. Murakami G. Mukai H. Hojo Y. Saito M. (2012). Corticosterone induces rapid spinogenesis via synaptic glucocorticoid receptors and kinase networks in hippocampus. PLoS ONE 7:e34124 10.1371/journal.pone.0034124 22509272 PMC3324490

[B21] Komatsuzaki Y. Murakami G. Tsurugizawa T. Mukai H. Tanabe N. Mitsuhashi K. (2005). Rapid spinogenesis of pyramidal neurons induced by activation of glucocorticoid receptors in adult male rat hippocampus. Biochem. Biophys. Res. Commun. 335, 1002–1007 10.1016/j.bbrc.2005.07.173 16111661

[B22] Luine V. N. Jacome L. F. Maclusky N. J. (2003). Rapid enhancement of visual and place memory by estrogens in rats. Endocrinology 144, 2836–2844 10.1210/en.2003-0004 12810538

[B23] MacLusky N. J. Luine V. N. Hajszan T. Leranth C. (2005). The 17alpha and 17beta isomers of estradiol both induce rapid spine synapse formation in the CA1 hippocampal subfield of ovariectomized female rats. Endocrinology 146, 287–293 10.1210/en.2004-0730 15486220

[B24] Markham J. A. McKian K. P. Stroup T. S. Juraska J. M. (2005). Sexually dimorphic aging of dendritic morphology in CA1 of hippocampus. Hippocampus 15, 97–103 10.1002/hipo.20034 15390161

[B25] McCarthy M. M. (2008). Estradiol and the developing brain. Physiol. Rev. 88, 91–124 10.1152/physrev.00010.2007 18195084 PMC2754262

[B26] McIlwain H. B. H. (1985). Biochemistry and the Central Nervous System. Edinburgh: Churchill Livingstone

[B31] Mukai H. Hatanaka Y. Mitsuhashi K. Hojo Y. Komatsuzaki Y. Sato R. (2011). Automated analysis of spines from confocal laser microscopy images: application to the discrimination of androgen and estrogen effects on spinogenesis. Cereb. Cortex 21, 2704–2711 10.1093/cercor/bhr059 21527787 PMC3209797

[B32] Mukai H. Kimoto T. Hojo Y. Kawato S. Murakami G. Higo S. (2010). Modulation of synaptic plasticity by brain estrogen in the hippocampus. Biochim. Biophys. Acta 1800, 1030–1044 10.1016/j.bbagen.2009.11.002 19909788

[B33] Mukai H. Takata N. Ishii H. T. Tanabe N. Hojo Y. Furukawa A. (2006). Hippocampal synthesis of estrogens and androgens which are paracrine modulators of synaptic plasticity: synaptocrinology. Neuroscience 138, 757–764 10.1016/j.neuroscience.2005.09.010 16310315

[B34] Mukai H. Tsurugizawa T. Murakami G. Kominami S. Ishii H. Ogiue-Ikeda M. (2007). Rapid modulation of long-term depression and spinogenesis via synaptic estrogen receptors in hippocampal principal neurons. J. Neurochem. 100, 950–967 10.1111/j.1471-4159.2006.04264.x 17266735

[B35] Murakami G. Tsurugizawa T. Hatanaka Y. Komatsuzaki Y. Tanabe N. Mukai H. (2006). Comparison between basal and apical dendritic spines in estrogen-induced rapid spinogenesis of CA1 principal neurons in the adult hippocampus. Biochem. Biophys. Res. Commun. 351, 553–558 10.1016/j.bbrc.2006.10.066 17070772

[B36] Okamoto M. Hojo Y. Inoue K. Matsui T. Kawato S. McEwen B. S. (2012). Mild exercise increases dihydrotestosterone in hippocampus providing evidence for androgenic mediation of neurogenesis. Proc. Natl. Acad. Sci. U.S.A. 109, 13100–13105 10.1073/pnas.1210023109 22807478 PMC3420174

[B37] Ooishi Y. Mukai H. Hojo Y. Murakami G. Hasegawa Y. Shindo T. (2012). Estradiol rapidly rescues synaptic transmission from corticosterone-induced suppression via synaptic/extranuclear steroid receptors in the hippocampus. Cereb. Cortex 22, 926–936 10.1093/cercor/bhr164 21725036

[B38] Pardridge W. M. Mietus L. J. (1979). Transport of steroid hormones through the rat blood-brain barrier. Primary role of albumin-bound hormone. J. Clin. Invest. 64, 145–154 10.1172/JCI109433 447850 PMC372100

[B40] Rapp P. R. Morrison J. H. Roberts J. A. (2003). Cyclic estrogen replacement improves cognitive function in aged ovariectomized rhesus monkeys. J. Neurosci. 23, 5708–5714 12843274 10.1523/JNEUROSCI.23-13-05708.2003 PMC6741262

[B41] Sandstrom N. J. Williams C. L. (2004). Spatial memory retention is enhanced by acute and continuous estradiol replacement. Horm. Behav. 45, 128–135 10.1016/j.yhbeh.2003.09.010 15019800

[B42] Sinopoli K. J. Floresco S. B. Galea L. A. (2006). Systemic and local administration of estradiol into the prefrontal cortex or hippocampus differentially alters working memory. Neurobiol. Learn. Mem. 86, 293–304 10.1016/j.nlm.2006.04.003 16730465

[B43] Spencer J. L. Waters E. M. Milner T. A. McEwen B. S. (2008). Estrous cycle regulates activation of hippocampal Akt, LIM kinase, and neurotrophin receptors in C57BL/6 mice. Neuroscience 155, 1106–1119 10.1016/j.neuroscience.2008.05.049 18601981 PMC2621322

[B44] Tanford C. Nozaki Y. Reynolds J. A. Makino S. (1974). Molecular characterization of proteins in detergent solutions. Biochemistry 13, 2369–2376 10.1021/bi00708a021 4364776

[B45] Tang Y. Janssen W. G. Hao J. Roberts J. A. McKay H. Lasley B. (2004). Estrogen replacement increases spinophilin-immunoreactive spine number in the prefrontal cortex of female rhesus monkeys. Cereb. Cortex 14, 215–223 10.1093/cercor/bhg121 14704219

[B46] Tsurugizawa T. Mukai H. Tanabe N. Murakami G. Hojo Y. Kominami S. (2005). Estrogen induces rapid decrease in dendritic thorns of CA3 pyramidal neurons in adult male rat hippocampus. Biochem. Biophys. Res. Commun. 337, 1345–1352 10.1016/j.bbrc.2005.09.188 16242668

[B47] Vierk R. Glassmeier G. Zhou L. Brandt N. Fester L. Dudzinski D. (2012). Aromatase inhibition abolishes LTP generation in female but not in male mice. J. Neurosci. 32, 8116–8126 10.1523/JNEUROSCI.5319-11.2012 22699893 PMC6703647

[B48] Walf A. A. Rhodes M. E. Frye C. A. (2006). Ovarian steroids enhance object recognition in naturally cycling and ovariectomized, hormone-primed rats. Neurobiol. Learn. Mem. 86, 35–46 10.1016/j.nlm.2006.01.004 16529958 PMC3625951

[B49] Warren S. G. Humphreys A. G. Juraska J. M. Greenough W. T. (1995). LTP varies across the estrous cycle: enhanced synaptic plasticity in proestrus rats. Brain Res. 703, 26–30 10.1016/0006-8993(95)01059-9 8719612

[B50] Warren S. G. Juraska J. M. (1997). Spatial and nonspatial learning across the rat estrous cycle. Behav. Neurosci. 111, 259–266 10.1037/0735-7044.111.2.259 9106666

[B51] Watanabe G. Taya K. Sasamoto S. (1990). Dynamics of ovarian inhibin secretion during the oestrous cycle of the rat. J. Endocrinol. 126, 151–157 10.1677/joe.0.1260151 2116492

[B52] Woolley C. S. Gould E. Frankfurt M. McEwen B. S. (1990). Naturally occurring fluctuation in dendritic spine density on adult hippocampal pyramidal neurons. J. Neurosci. 10, 4035–4039 2269895 10.1523/JNEUROSCI.10-12-04035.1990 PMC6570039

[B53] Woolley C. S. McEwen B. S. (1992). Estradiol mediates fluctuation in hippocampal synapse density during the estrous cycle in the adult rat. J. Neurosci. 12, 2549–2554 1613547 10.1523/JNEUROSCI.12-07-02549.1992 PMC6575846

[B54] Woolley C. S. McEwen B. S. (1993). Roles of estradiol and progesterone in regulation of hippocampal dendritic spine density during the estrous cycle in the rat. J. Comp. Neurol. 336, 293–306 10.1002/cne.903360210 8245220

[B55] Xie X. S. Stone D. K. (1986). Isolation and reconstitution of the clathrin-coated vesicle proton translocating complex. J. Biol. Chem. 261, 2492–2495 2869030

[B56] Yildirim M. Janssen W. G. Tabori N. E. Adams M. M. Yuen G. S. Akama K. T. (2008). Estrogen and aging affect synaptic distribution of phosphorylated LIM kinase (pLIMK) in CA1 region of female rat hippocampus. Neuroscience 152, 360–370 10.1016/j.neuroscience.2008.01.004 18294775 PMC2396523

[B57] Znamensky V. Akama K. T. McEwen B. S. Milner T. A. (2003). Estrogen levels regulate the subcellular distribution of phosphorylated Akt in hippocampal CA1 dendrites. J. Neurosci. 23, 2340–2347 12657693 10.1523/JNEUROSCI.23-06-02340.2003 PMC6742003

