# Supplementary material for: Female hippocampal estrogens have a significant correlation with cyclic fluctuation of hippocampal spines
Source: Front Neural Circuits. 2013 Oct 18;7:149. doi: 10.3389/fncir.2013.00149 (PMC3798982; doi:10.3389/fncir.2013.00149)
Supplement: Figure S1 — Steroid derivatives and their fragmented ions used for analysis with LC-MS/MS. (A) PROG (m/z = 315) and its fragmented ion (m/z = 97), (B) E1-3-picolinoyl-ester (m/z = 376) and its fragmented ion (m/z = 157), (C) E2-3-pentafluorobenzoxy-17-picolinoyl-ester (m/z = 558) and its fragmented ion (m/z = 339), (D) ADione (m/z = 287) and its fragmented ion (m/z = 109), (E) T-17-picolinoyl-ester (m/z = 394) and its fragmented ion (m/z = 253). Picolinoyl-ester is used for induced-inonization, and pentafluorobenzoxy-ester is particularly used to increase evaporation probability of E2. Note that PROG and ADione were measured without derivatization. [file Presentation1.PDF]

Fig.S1

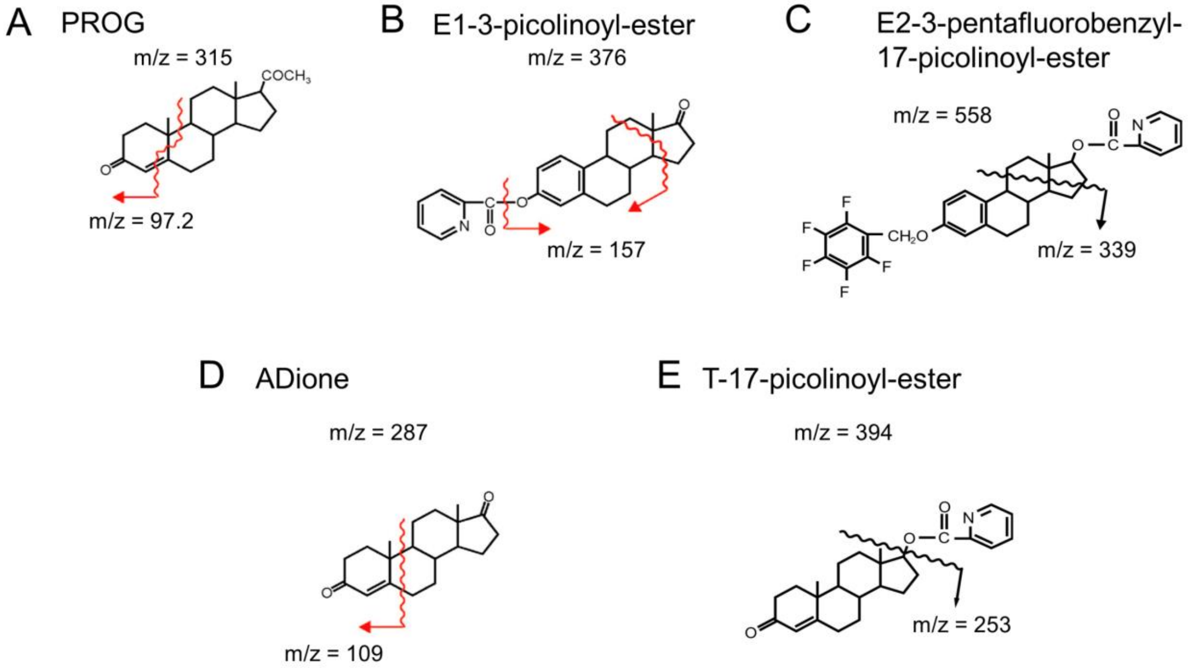

A PROG
m/z = 315
$COCH_3$
m/z = 97.2
B E1-3-picolinoyl-ester
m/z = 376
m/z = 157
C E2-3-pentafluorobenzyl-17-picolinoyl-ester
m/z = 558
CH_2O
F
m/z = 339
D ADione
m/z = 287
m/z = 109
E T-17-picolinoyl-ester
m/z = 394
m/z = 253
