# Supplementary figures and images for: Female hippocampal estrogens have a significant correlation with cyclic fluctuation of hippocampal spines
Source: Front Neural Circuits. 2013 Oct 18;7:149. doi: 10.3389/fncir.2013.00149 (PMC3798982; doi:10.3389/fncir.2013.00149)

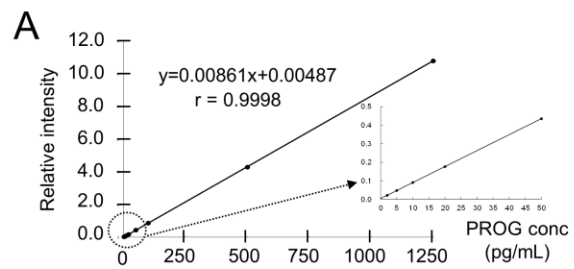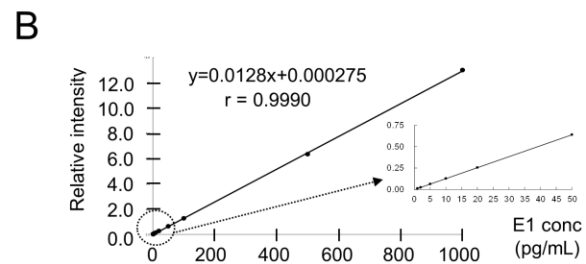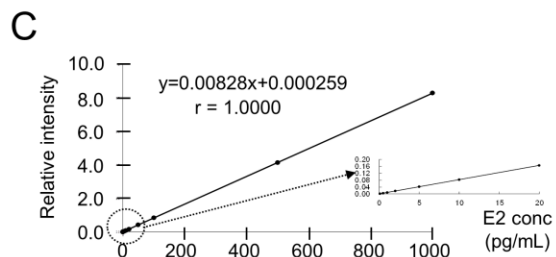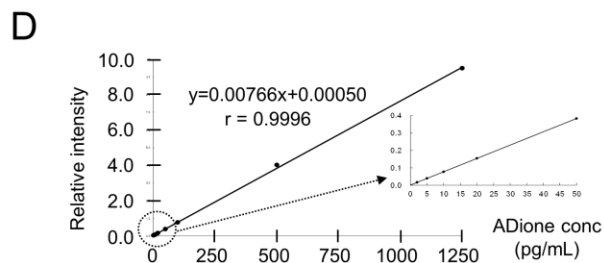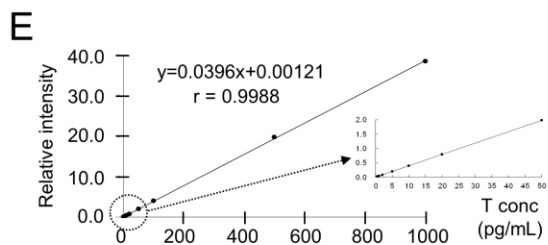

Fig.S2

Supplement: Figure S2 — Calibration curves for LC-MS/MS using standard steroids dissolved in ethanol. Horizontal (x) axis indicates the concentration of added standard steroid. Vertical (y) axis indicates the relative intensity obtained from the chromatogram. (A) Calibration curve for PROG. Linearity was observed between 2 pg/mL and 1250 pg/mL. (B) Calibration curve for E1. Linearity was observed between 1 pg/mL and 1000 pg/mL. (C) Calibration curve for E2. Linearity was observed between 0.1 and 1000 pg/mL. (D) Calibration curve for ADione. Linearity was observed between 2 and 1250 pg/mL. (E) Calibration curve for T. Linearity was observed between 0.5 and 1000 pg/mL. [file Presentation2.PDF]

Fig. S3

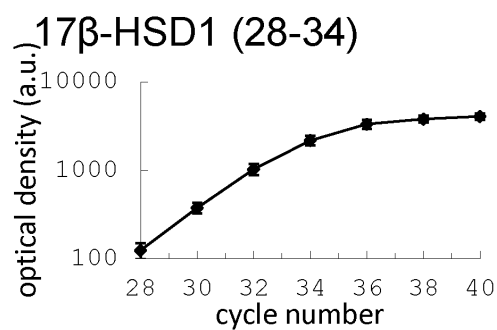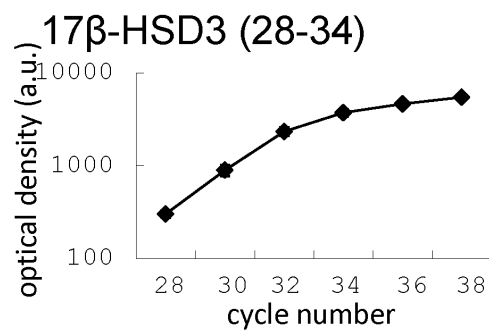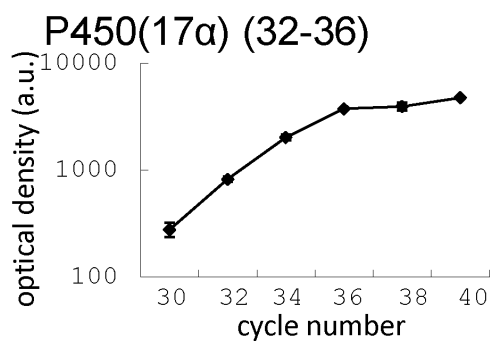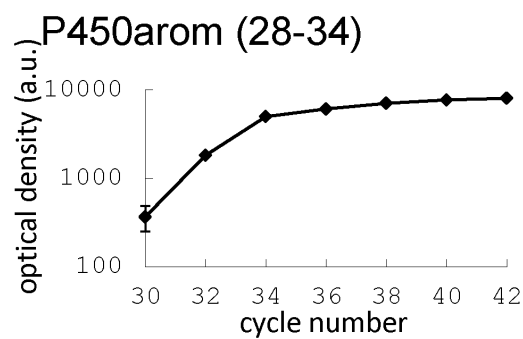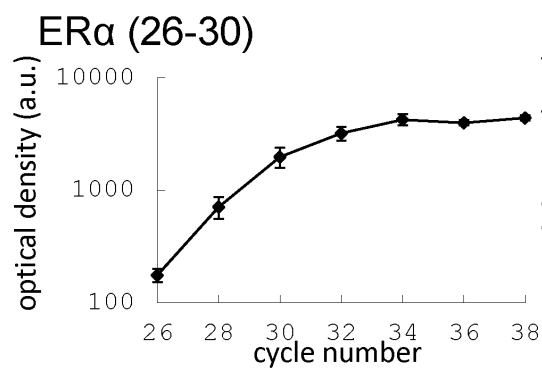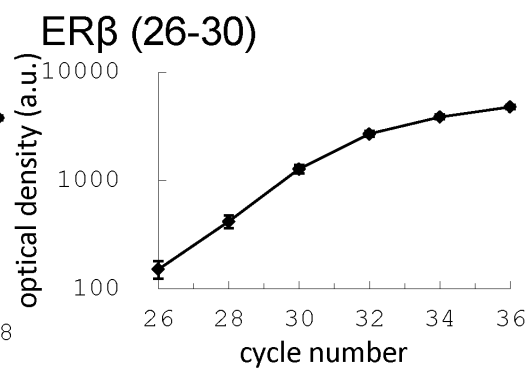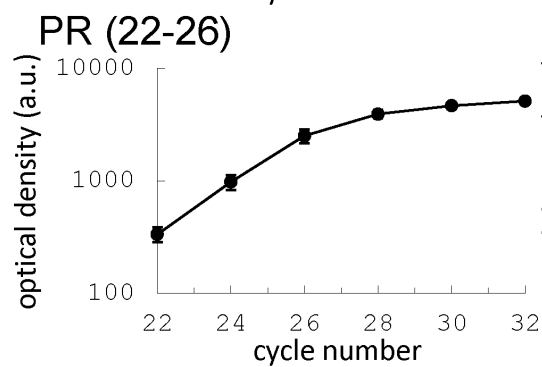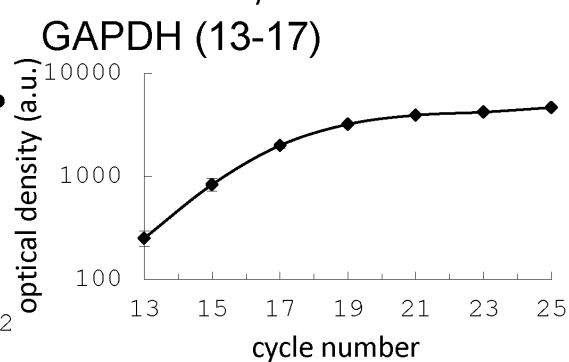

Supplement: Figure S3 — Amplification curves for steroidogenic enzymes and receptors. The PCR cycle numbers within linear phase are indicated in the parentheses. Each PCR amplification curve was obtained from cDNA made by reverse-transcription from 100 ng of hippocampal total RNA from the hippocampus. These cDNA templates were used for the experiments shown in Figures 3, 4. See also Table S2. [file Presentation3.PDF]
