# Supplementary material for: Female hippocampal estrogens have a significant correlation with cyclic fluctuation of hippocampal spines
Source: Front Neural Circuits. 2013 Oct 18;7:149. doi: 10.3389/fncir.2013.00149 (PMC3798982; doi:10.3389/fncir.2013.00149)
Supplement: Table S1 — The intra-and inter-assay of accuracy and precision as well as the limit of quantification (LOQ) for each steroid. [file DataSheet1.PDF]

**Table S1**

The intra- and inter-assay of accuracy and precision as well as the limit of quantification (LOQ) for each steroid.

| | m/z[b] transition | Spike (pg) | Intraassay ($n^a = 5$) Accuracy (%) | Intraassay RSD[c] (%) | Interassay ($n^a = 3$) Accuracy (%) | Interassay RSD (%) | LOQ[d] (pg/0.1g) |
|---|---|---|---|---|---|---|---|
| 17β-E2 | from 558 to 339 | 0.3 | 95.5 | 5.9 | 92.3 | 9.2 | 0.3 |
| | | 5 | 96.3 | 4.3 | 97.0 | 6.7 | |
| | | 20 | 101.1 | 2.3 | 101.0 | 1.3 | |
| T | from 394 to 253 | 1 | 108.2 | 3.9 | 97.3 | 4.3 | 1 |
| | | 10 | 93.6 | 2.1 | 98.2 | 2.1 | |
| | | 100 | 99.8 | 0.4 | 102.2 | 1.6 | |
| E1 | from 376 to 157 | 1 | 105.8 | 5.8 | 98.5 | 2.7 | 1 |
| | | 10 | 106.4 | 3.8 | 100.5 | 3.2 | |
| | | 100 | 99.8 | 1.0 | 99.2 | 3.1 | |
| PROG | from 315 to 97 | 2 | 108.1 | 6.3 | 105.3 | 4.8 | 2 |
| | | 10 | 104.8 | 3.6 | 97.3 | 4.1 | |
| | | 100 | 97.7 | 1.9 | 98.8 | 2.2 | |
| ADione | from 287 to 109 | 2 | 94.1 | 4.3 | 106.5 | 6.2 | 2 |
| | | 10 | 98.7 | 3.2 | 103.7 | 2.8 | |
| | | 100 | 99.6 | 1.9 | 104.8 | 2.1 | |

Blank samples, prepared alongside hippocampal samples through the whole extraction and purification procedures, were spiked with E2 or other steroids at 0.1, 0.3, 1, 2, 5,10, 20 and 100 pg, and contents were determined by LC-MS/MS. Accuracy was expressed as a percentage of an analytical recovery rate of measured steroid content against spike amount.

[a]For each condition, intra- and interassay were performed five and three times, respectively.

[b]m and z represent the mass and charge of a steroid derivative, respectively.

[c]relative standard deviation.

[d]LOQ is expressed as pg/0.1 g. Because the average weight of one whole adult hippocampus (0.14 g) was close to 0.1 g, these LOQ values indicate the limit of quantification of steroids from nearly one hippocampus.
