# Supplementary material for: Female hippocampal estrogens have a significant correlation with cyclic fluctuation of hippocampal spines
Source: Front Neural Circuits. 2013 Oct 18;7:149. doi: 10.3389/fncir.2013.00149 (PMC3798982; doi:10.3389/fncir.2013.00149)
Supplement: Table S2 — The sequence of primer oligonucleotides for PCR amplification. [file DataSheet2.PDF]

Table S2

The sequence of primer oligonucleotides for PCR amplification.

target mRNA		sequence (5' → 3')	product length(bp)[a]	T_a(°C)[b]	PCR cycles[c]
17β-HSD			517		
(type1)	Forward	ACTCCGGGCGTGTGCTGGTGA		65	33
	Reverse	GGCGTGTCTGGATCCCCTGAAACTT			(28-34)
17β-HSD			408		
(type3)	Forward	CTCCCCAACCTGCTCCCAAGTCATTT		65	34
	Reverse	AGCAAGGCAGCCACAGGTTTCAGC			(28-34)
P450(17α)	Forward	TGGGGCGGCATAGAGACAACT	477	62	36
	Reverse	AGCAAGGCCGTGAAGACAAAGAGC			(32-36)
P450(arom)	Forward	CTGATCATGGGCCTCCTCCTG	276	58	34
	Reverse	CCCACGCTTGCTGCCGAATCT			(28-34)
ERα	Forward	GCCGGCTGCGCAAGTGTTACG	467	68	30
	Reverse	GGAGCGCCAGACCAGACCAATCA			(26-30)
ERβ	Forward	GCAAACCAGGAGGCAGAAAGTAGC	591	58	30
	Reverse	AAGTGGGCAAGGAGACAGAAAGTAAGTA			(26-30)
PR	Forward	TGAATGAGCAGAGGATGAAGGAGTTG	353	60	25
	Reverse	CAGTGCCCGGGATTGGATG			(22-26)
GAPDH	Forward	TATGACTCTACCCACGGCAAGTTCAA	830	60	17
	Reverse	ACCACCCTGTTGCTGTAGCCATATTCAT			(13-17)

a bp : base pair.

b Ta : annealing temperature.

c upper column: PCR cycles used for semi-quantitative analysis.
lower column: PCR cycles within linear phase in amplification curve.
