# Supplementary material for: Female hippocampal estrogens have a significant correlation with cyclic fluctuation of hippocampal spines
Source: Front Neural Circuits. 2013 Oct 18;7:149. doi: 10.3389/fncir.2013.00149 (PMC3798982; doi:10.3389/fncir.2013.00149)
Supplement: Table S3 — The accuracy of steroid determination for hippocampal tissue spiked with exogenous steroids. [file DataSheet3.PDF]

Table S3

The accuracy of steroid determination for hippocampal tissue spiked with exogenous steroids.

	Steroid	Added (ng/g)	Found (ng/g)	Accuracy (%)[a]
Hippocampus	E2	0.0	2.302	-
		0.5	2.838 (0.536)[b]	107.2
		1.0	3.349 (1.047)[b]	104.7
	T	0.0	5.316	-
		0.5	5.827 (0.511)[b]	102.2
		1.0	6.308 (0.998)[b]	99.8
	E1	0.0	0.004	-
		0.5	4.578 (4.574)[b]	91.5
		1.0	4.671 (4.667)[b]	93.3
	ADione	0.0	0.444	-
		0.5	0.989 (0.545)[b]	108.8
		1.0	1.476 (1.032)[b]	103.2
	PROG	0.0	4.911	-
		0.5	5.384 (0.473)[b]	94.6
		1.0	5.895 (0.984)[b]	98.4

[a] Accuracy was expressed as a recovery rate (%) of the measured amount of steroid to the added amount of steroid.

[b] Value in parentheses was obtained by subtraction of the endogenous amount of steroid from the value obtained for each spiked steroid.
